# Methionine Restriction Extends Yeast Lifespan by Activating Non‐Nitrogen‐Starvation‐Induced Autophagy Through Limiting Methylation of Protein Phosphatase 2A

**DOI:** 10.1111/acel.70550

**Published:** 2026-05-20

**Authors:** Kaylah Birmingham, Nina Arslanovic, Thea Grauer, Ignacio Gutierrez, Ujani Chakraborty, Simone Sidoli, Jessica Tyler

**Affiliations:** ^1^ Department of Pathology and Laboratory Medicine Weill Cornell Medicine New York New York USA; ^2^ Pharmacology Graduate Program Weill Cornell Medicine New York New York USA; ^3^ Department of Biochemistry Albert Einstein College of Medicine New York New York USA

**Keywords:** autophagy, lifespan, longevity, methionine restriction, methylation, phosphorylation

## Abstract

Methionine restriction (MR) extends the lifespan and healthspan of numerous eukaryotic organisms, but the molecular mechanisms at play are unclear. Here we find that the ability of MR to extend the budding yeast chronological and replicative lifespans is the consequence of reduced methionine conversion to the methyl donor S‐adenosylmethionine (SAM). Mechanistically, the key antiaging event downregulated by MR is the methylation of protein phosphatase 2A (PP2A). In chronological aging cells under MR, unmethylated PP2A no longer dephosphorylates Npr2, a component of the SEACIT complex, resulting in activation of non‐nitrogen‐starvation (NNS)‐induced autophagy. Deletion of genes encoding components of SEACIT or *ATG1* (encoding a central player in the initiation of autophagy) blocked the ability of MR to extend lifespan, showing the critical role of the NNS‐induced autophagy pathway in lifespan extension by MR. We identify the relevant Npr2 site dephosphorylated by PP2A as serine 362 and show that Npr2 phosphomimetic mutants are sufficient to extend chronological and replicative lifespan. Finally, we discover that MR only during the early stages of chronological aging is sufficient to prolong autophagy and extend lifespan. In addition to elucidating the molecular mechanism of MR‐mediated lifespan extension, this study highlights potential therapeutic targets to achieve lifespan and healthspan extension in humans without the challenging long‐term dietary changes required to achieve MR.

## Graphical abstract

Methionine restriction extends yeast replicative and chronological lifespan as a consequence of depletion of S‐adenosylmethionine (SAM). SAM depletion leads to demethylation of PP2A and increased phosphorylation of Npr2 which activates non‐nitrogen‐starvation (NNS) induced autophagy; early methionine restriction is sufficient to activate persistent autophagy and lifespan extension, implicating an epigenetic memory.

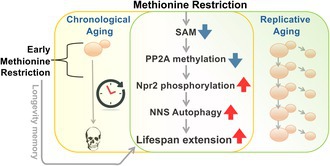

## 1 Introduction

Research using model organisms has uncovered molecular insights into cellular aging relevant to humans. *Saccharomyces cerevisiae* is a particularly powerful model for lifespan research as many of the key biological pathways of aging in mammals are conserved in yeast. For example, most of the known anti‐aging interventions, such as calorie restriction, extend lifespan in mammals and yeast, presumably functioning through conserved fundamental pathways (Kennedy et al. 2007). Furthermore, budding yeast enables the study of two separate models of aging. Yeast chronological lifespan (CLS), which models the aging of post‐mitotic cells in higher eukaryotes, and is measured by determining the length of time non‐dividing cells remain viable (Fabrizio and Longo 2003). On the other hand, yeast replicative lifespan (RLS) models mitotic aging and is measured by the number of divisions a given cell undergoes before dying (Fabrizio and Longo 2007). Notably, budding yeast is the only type of eukaryotic cell that enables accurate measurement of the replicative lifespan. Research using the yeast CLS and RLS provides important insights into the conserved mechanisms of aging, relevant to humans.

Calorie restriction (CR) is a means to extend lifespan and healthspan (Fontana et al. 2010). This involves reducing food intake without malnutrition, but selecting the optimal regimen for CR is complicated, as the same regimen can lead to different outcomes in metazoans depending on various factors, the genotype and environmental factors. Also, the severity of the CR regimen required for optimal lifespan extension is unlikely to be feasible outside of lab settings and may have detrimental consequences for humans (Speakman and Hambly 2007). Diets with decreased levels of proteins or individual amino acids, such as methionine, support longevity without decreasing calorie intake, and may avoid the possible undesirable effects of CR in humans and the risk of malnutrition. Studies have indicated that decreased methionine intake may be responsible for the lifespan extension effect of CR (Miller et al. 2005; Orentreich et al. 1993). Indeed, methionine restriction (MR) extends the lifespan of many organisms, including rats and mice (Miller et al. 2005; Orentreich et al. 1993). In addition to its lifespan promoting effects, MR also improves glycemic control, enhances metabolic health, and reduces fatty liver disease in mammals (Miller et al. 2005; Plummer et al. 2021). Despite these well‐established beneficial health outcomes, the fundamental molecular mechanism of MR‐mediated lifespan extension is still largely unknown. Furthermore, MR in humans is difficult to achieve because methionine is in every protein, highlighting the importance for discovery of the key molecular changes induced by MR to extend lifespan as it will reveal therapeutic targets for anti‐aging treatments.

As the initiator of polypeptide synthesis, methionine is a vital contributor to translation of all proteins. Methionine also makes important contributions to other cellular processes, and it is unclear whether it is its role in protein synthesis or one of these other processes that is critical for the ability of MR to extend lifespan. As methionine gets processed, the metabolites produced are also crucial to various cellular processes. For example, methionine gets converted into S‐adenosylmethionine (SAM). Decarboxylated SAM can be used for synthesis of polyamines—compounds intricately involved in many cellular processes such as cell growth, cell death, and protein synthesis (Eisenberg et al. 2009; Thomas and Surdin‐Kerjan 1997). SAM is also the methyl donor used by methyltransferases; through SAM being converted to S‐adenosylhomocysteine (SAH), it can play roles in various methylation‐controlled events. Additionally, methionine products eventually get shuttled through the transsulfuration pathway generating metabolites involved in antioxidant defense and maintaining redox balance (Thomas and Surdin‐Kerjan 1997). It will be important to discern which of these pathways is the critical one downregulated by MR to promote lifespan extension.

Important insights into the mechanism of MR‐mediated lifespan extension came from the discovery that MR induces autophagy during the yeast CLS, where autophagy is the process used by the cell to recycle damaged cellular components (Ruckenstuhl et al. 2014). Autophagy has been implicated in anti‐aging in many model organisms (Gelino and Hansen 2012). The ability of the Tor1 inhibitor rapamycin to extend the yeast CLS depended on autophagy (Alvers et al. 2009). Moreover, the ability of MR to extend the yeast CLS was dependent on autophagy (Ruckenstuhl et al. 2014). How MR induces autophagy during aging was unknown. Here we show that the critical anti‐aging consequence of lower methionine levels was reduced SAM synthesis, leading to less methylation of protein phosphatase 2 (PP2A). During CLS, the unmethylated PP2A was unable to dephosphorylate serine 362 of the Npr2 component of the SEACIT complex which activated non‐nitrogen‐starvation (NNS)‐induced autophagy to extend chronological lifespan. We show that MR additionally extends the yeast replicative lifespan in a manner that is also dependent on reduced SAM levels, Npr2, and autophagy.

## 2 Methods

### 2.1 Strains and Plasmids

For all experiments, the strains were constructed in either the BY4741 parental strain (for all MR cultures during CLS as it is *met15∆*) or in the BY4742 parental strain of the S288C background (Brachmann et al. 1998) (Table 1). Homologous recombination was used to delete genes using PCR products from pFA6a‐KanMX and pFA6a‐HygroMX plasmid templates (Longtine et al. 1998). GFP‐Atg8 strains express Atg8 fused at the N‐terminus to GFP via integration of the GFP‐ATG8(416)/GFP‐AUT7(416) plasmid into the genome of the parental strain (Nair et al. 2011). Npr2‐Myc strains express a C‐terminal 13xMyc tag amplified from the plasmid pFA6a‐13Myc‐His3MX6. Phosphomutant strains were generated using CRISPR‐Cas‐9 mediated genome editing (Aguilar et al. 2022).

**TABLE 1:** *S. cerevisiae* strains used in this study.

Abbreviated name	Strain name	Genotype	Source
MR^a^	BY4741	*MATa his3Δ1 leu2Δ0 met15Δ0 ura3Δ0*	Euroscarf
Control	BY4742	*MATα his3Δ1 leu2Δ0 lys2Δ0 ura3Δ0*	Euroscarf
*met15∆*	KBY191	*MATα his3Δ1 leu2Δ0 lys2Δ0 ura3Δ0MET15::hphmx*	This study
*met2∆*	KBY036	*MATα his3Δ1 leu2Δ0 lys2Δ0 ura3Δ0 MET2::hphMX*	This study
*atg1∆*	KBY013	*MATα his3Δ1 leu2Δ0 lys2Δ0 ura3Δ0 ATG1::hphMX*	This study
*atg1∆* (MR)	KBY193	*MATa his3Δ1 leu2Δ0 met15Δ0 ura3Δ0 ATG1::hphMX*	This study
*GFP‐Atg8*	KBY066	*MATα his3Δ1 leu2Δ0 lys2Δ0 ura3Δ0 GFP‐ATG8*	This study
*atg1∆ GFP‐ATG8*	KBY067	*MATα his3Δ1 leu2Δ0 lys2Δ0 ura3Δ0 ATG1Δ::hphMx GFP‐ATG8*	This study
*GFP‐Atg8* (MR)	KBY063	*MATα his3Δ1 leu2Δ0 lys2Δ0 ura3Δ0 MET15∆::kanMX GFP‐ATG8*	This study
*atg1∆ GFP‐Atg8* (MR)	KBY057	*MATα his3Δ1 leu2Δ0 lys2Δ0 ura3Δ0 ATG1Δ::hphMx MET15∆::kanMX GFP‐ATG8*	This study
*npr2∆ GFP‐Atg8*	KBY139	*MATα his3Δ1 leu2Δ0 lys2Δ0 ura3Δ0 NPR2∆::kanMX GFP‐ATG8*	This study
*npr2∆ GFP‐Atg8* (MR)	KBY147	*MATα his3Δ1 leu2Δ0 lys2Δ0 ura3Δ0 NPR2∆::kanMX MET15∆::hphMX GFP‐ATG8*	This study
*Npr2‐Myc*	KBY124	*MATα his3Δ1 leu2Δ0 lys2Δ0 ura3Δ0 NPR2‐ MYC::HIS*	This study
*Npr2‐Myc* (MR)	KBY125	*MATa his3Δ1 leu2Δ0 met15Δ0 ura3Δ0 NPR2‐MYC::HIS*	This study
*ppm1∆*	KBY040	*MATα his3Δ1 leu2Δ0 lys2Δ0 ura3Δ0 PPM1∆::hphMX*	This study
*ppm1∆* (MR)	KBY068	*MATa his3Δ1 leu2Δ0 met15Δ0 ura3Δ0 PPM1∆::hphMX*	This study
*rph1∆*	KBY044	*MATα his3Δ1 leu2Δ0 lys2Δ0 ura3Δ0 RPH1∆::hphMX*	This study
*rph1∆* (MR)	KBY042	*MATa his3Δ1 leu2Δ0 met15Δ0 ura3Δ0 RPH1∆::hphMX*	This study
*rph1 met2∆*	KBY089	*MATα his3Δ1 leu2Δ0 lys2Δ0 ura3Δ0 RPH1∆::hphMX MET2::kanMX*	This study
*npr2∆*	KBY030	*MATα his3Δ1 leu2Δ0 lys2Δ0 ura3Δ0 NPR2∆::kanMX*	This study
*npr2∆* (MR)	KBY045	*MATa his3Δ1 leu2Δ0 met15Δ0 ura3Δ0 NPR2∆::kanMX*	This study
*npr2∆ met2∆*	KBY050	*MATα his3Δ1 leu2Δ0 lys2Δ0 ura3Δ0 NPR2∆::kanMX MET2∆::kanMX*	This study
*npr3∆*	KBY058	*MATα his3Δ1 leu2Δ0 lys2Δ0 ura3Δ0 NPR3∆::hphMX*	This study
*npr3∆* (MR)	KBY061	*MATa his3Δ1 leu2Δ0 met15Δ0 ura3Δ0 NPR3∆::hphMX*	This study
*iml1∆*	KBY086	*MATα his3Δ1 leu2Δ0 lys2Δ0 ura3Δ0 IML1∆::hphMX*	This study
*iml1∆* (MR)	KBY083	*MATa his3Δ1 leu2Δ0 met15Δ0 ura3Δ0 IML1∆::hphMX*	This study
*cdc55∆*	KBY127	*MATα his3Δ1 leu2Δ0 lys2Δ0 ura3Δ0 NPR2‐ MYC::HIS CDC55∆::hphMX*	This study
*rts1∆*	KBY143	*MATα his3Δ1 leu2Δ0 lys2Δ0 ura3Δ0 NPR2‐ MYC::HIS RTS1∆::kanMX*	This study
*NPR2 S362A*	KBY072	*MATα his3Δ1 leu2Δ0 lys2Δ0 ura3Δ0 NPR2‐S362A*	This study
*NPR2 S362E*	KBY073	*MATα his3Δ1 leu2Δ0 lys2Δ0 ura3Δ0 NPR2‐S362E*	This study
*NPR2 S362D*	KBY074	*MATα his3Δ1 leu2Δ0 lys2Δ0 ura3Δ0 NPR2‐S362D*	This study
*NPR2 S362A* (MR)	KBY075	*MATα his3Δ1 leu2Δ0 lys2Δ0 ura3Δ0 NPR2‐S362A MET15:: hphmx*	This study
*NPR2 S362E* (MR)	KBY077	*MATα his3Δ1 leu2Δ0 lys2Δ0 ura3Δ0 NPR2‐S362E MET15:: hphmx*	This study
*NPR2 S362D* (MR)	KBY071	*MATa his3Δ1 leu2Δ0 met15Δ0 ura3Δ0 NPR2‐S362D*	This study
*NPR2 S362A met2∆*	KBY103	*MATα his3Δ1 leu2Δ0 lys2Δ0 ura3Δ0 NPR2‐S362A MET2:: hphmx*	This study
*NPR2 S362E met2∆*	KBY106	*MATα his3Δ1 leu2Δ0 lys2Δ0 ura3Δ0 NPR2‐S362E MET2:: hphmx*	This study
*NPR2 S362D met2∆*	KBY109	*MATα his3Δ1 leu2Δ0 lys2Δ0 ura3Δ0 NPR2‐S362D MET2:: hphmx*	This study
*tor1∆*	KBY081	*MATα his3Δ1 leu2Δ0 lys2Δ0 ura3Δ0 TOR1∆:: kanMX*	This study
*tor1∆* (MR)	KBY079	*MATa his3Δ1 leu2Δ0 met15Δ0 ura3Δ0 TOR1∆::kanMX*	This study
*NPR2 S362A GFP‐Atg8*	KBY164	*MATα his3Δ1 leu2Δ0 lys2Δ0 ura3Δ0 NPR2‐S362A GFP‐ATG8*	This study
*NPR2 S362A GFP‐Atg8* (MR)	KBY167	*MATα his3Δ1 leu2Δ0 lys2Δ0 ura3Δ0 NPR2‐S362A MET15:: hphmx GFP‐ATG8*	This study
*NPR2 S362E GFP‐Atg8*	KBY170	*MATα his3Δ1 leu2Δ0 lys2Δ0 ura3Δ0 NPR2‐S362E GFP‐ATG8*	This study
*NPR2 S362E GFP‐Atg8* (MR)	KBY173	*MATα his3Δ1 leu2Δ0 lys2Δ0 ura3Δ0 NPR2‐S362E MET15:: hphmx GFP‐ATG8*	This study
*NPR2 S362D GFP‐Atg8*	KBY176	*MATα his3Δ1 leu2Δ0 lys2Δ0 ura3Δ0 NPR2‐S362D GFP‐ATG8*	This study
*NPR2 S362D GFP‐Atg8* (MR)	KBY179	*MATα his3Δ1 leu2Δ0 lys2Δ0 ura3Δ0 NPR2‐S362D MET15:: hphmx GFP‐ATG8*	This study

^a^ Because most strains were made in BY4741 and BY4742, all BY4741 strains have been named denoted by “MR” in the strain name.

### 2.2 Lifespan Assays

CLS experiments were started by inoculating single colonies into 3 mL of liquid synthetic complete (SC) media (0.67% yeast nitrogen base (YNB) without amino acids, 0.45% casamino acids (contains an unspecified amount of methionine), 2% glucose, 0.01% tryptophan, 0.008% adenine sulfate, and 0.009% uridine) as previously described (Plummer et al. 2021) in 15 mL tubes that were rotated at 30°C for 72 h. Optical densities (OD) at 600 nm were taken and the cultures were diluted to OD of 0.2 in fresh SC media, and this time was designated time = 0 of the CLS. Every day for the first 3 days of growth, 10 μL aliquots were removed, serially diluted at 1:10,000, and then plated on YPD plates to assess their colony forming units (CFU). Once a maximum CFU value was reached, aliquots were plated every 48 h. RLS assays were performed in SC media (0.17% YNB without amino acids and ammonium sulfate, 0.2% drop‐out mix complete (supplies 32.4 mg/liter methionine) without YNB, 0.5% ammonium sulfate, and 2% glucose, as previously described (Gutierrez and Tyler 2024)). *p*‐values were calculated using the Log‐rank method. Calorie Restriction was performed with SC medium using 0.05% glucose and 100 mM sorbitol instead of 2% glucose.

### 2.3 Mass Spectrometry of Histone Methylation

Histones were isolated according to published methods (Fukuma et al. 1994). The mass spectrometry was performed as described previously (Stransky et al. 2023). Histone peptides raw files were imported into the EpiProfile 2.0 software (Yuan et al. 2018). The resulting peptide lists generated by EpiProfile were exported to Microsoft Excel and further processed for a detailed analysis.

### 2.4 Western Blots

Total and unmethylated PP2A was measured as described previously (Ye et al. 2019), using anti‐PP2A (Millipore 05–545) and anti‐G6PDH (Abcam ab8245). Npr2 phosphoshift western blots used: anti‐Myc (Millipore M4439), anti‐G6PDH (Abcam ab8245). Release of free GFP from Atg8 was measured as we described previously (Plummer et al. 2021) using anti‐GFP (Abcam Ab290) and anti‐G6PDH (Sigma‐Aldrich A9521). Histone methylation western blots used anti‐H3 (Abcam ab1791), anti‐H3K36me3 (Abcam ab9050), and anti‐GAPDH (Abcam ab8245).

## 3 Results

### 3.1 Supplementation With SAM, but Not SAH, Prevents CLS Extension by MR

**FIGURE 1:**
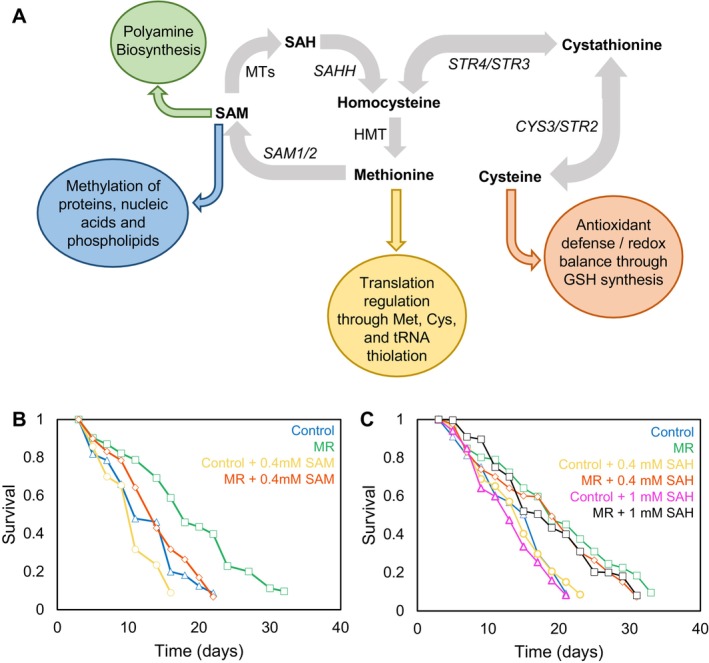
Addition of SAM, but not SAH, prevents lifespan extension by MR. (A) Methionine metabolism and the coupling of methionine and its derivatives to various cellular functions. *CYS3*, cystathionine gamma‐lyase; HMT, histone methyltransferase; MTS, methyltransferases; *SAHH*, SAH hydrolase; *SAM1/2*, SAM synthetase 1 & 2; *STR2*, cystathionine gamma‐synthase; *STR3*, cystathionine beta‐synthase; *STR4*, peroxisomal cystathionine beta‐lyase. (B) CLS of Control and MR (*met15∆*) yeast strains +/− SAM (400 μM) and (C) +/− 1 mM SAH.

To differentiate between the role of methionine in protein synthesis and its role as a precursor for SAM synthesis driving MR‐mediated lifespan extension (Figure 1A), we added SAM to yeast undergoing MR to see if it prevented lifespan extension. We modeled MR in yeast by using the BY4741 deletion collection yeast strain, which lacks *MET15*—a gene that encodes a key methionine biosynthesis enzyme (Brachmann et al. 1998). The *met15* strain is a more consistent approach to achieve MR than dietary MR (Johnson and Johnson 2014). Genetic methionine restriction in yeast leads to a reduction of both intracellular methionine and SAM levels (Fang et al. 2023). For the methionine replete control, we used the *MET15* strain BY4742. The difference in mating type between the two strains has no effect on lifespan, as deletion of *MET15* in BY4742 increased chronological lifespan (CLS) similarly to BY4741 (Figure S1A). Importantly, addition of SAM prevented CLS extension by MR (Figure 1B). Of note, addition of SAM did not prevent all CLS extension by antiaging interventions, as calorie restricted (CR) yeast were still able to live longer in the presence of SAM supplementation (Figure S1B). These results demonstrate that MR, but not CR, promotes CLS extension in a manner dependent on reduced SAM production as opposed to reduced protein synthesis.

SAM functions as the methyl donor for methylation of proteins, nucleic acids, and lipids, as well as in the synthesis of polyamines (Figure 1A). SAM is also the precursor to SAH, which promotes antioxidant defense and redox balance (Figure 1A). To determine if SAM depletion functions upstream or downstream of SAH production to extend lifespan, we added SAH to yeast undergoing MR to see if it prevented lifespan extension. SAH did not reduce CLS extension by MR—even at 1 mM, a concentration more than double that which blocks MR‐dependent lifespan extension by SAM (Figure 1B,C). These results demonstrate that it is the depletion of SAM, not SAH, that is responsible for MR‐mediated CLS extension.

### 3.2 MR Does Not Reduce Histone Methylation

Given MR's reliance on reduced SAM levels to elicit its antiaging effect (Figure 1) and the influence of histone methylation on lifespan in other organisms (Yi and Kim 2020), we asked whether MR was working through reducing histone methylation. To address this question, we performed LC–MS/MS to determine the relative abundance of each modified/unmodified histone (Fukuma et al. 1994) on both Day 0 and Day 3 of the CLS with and without MR. In contrast to the histone demethylation that is observed upon methionine starvation (Ye et al. 2019), histone demethylation was not apparent at Day 3 of the CLS during MR (Figure S2A & Table S1). Counterintuitively, H3K36 trimethylation was increased in MR cells on Day 3 of the CLS compared to control cells (Figure S2A), as confirmed by western blotting (Figure S2B). This suggests that there may be selective regulation of H3K36 methyltransferases or demethylases rather than a global effect due to low SAM availability during MR conditions (see later discussions).

### 3.3 MR Activates NNS‐Induced Autophagy to Extend Lifespan

**FIGURE 2:**
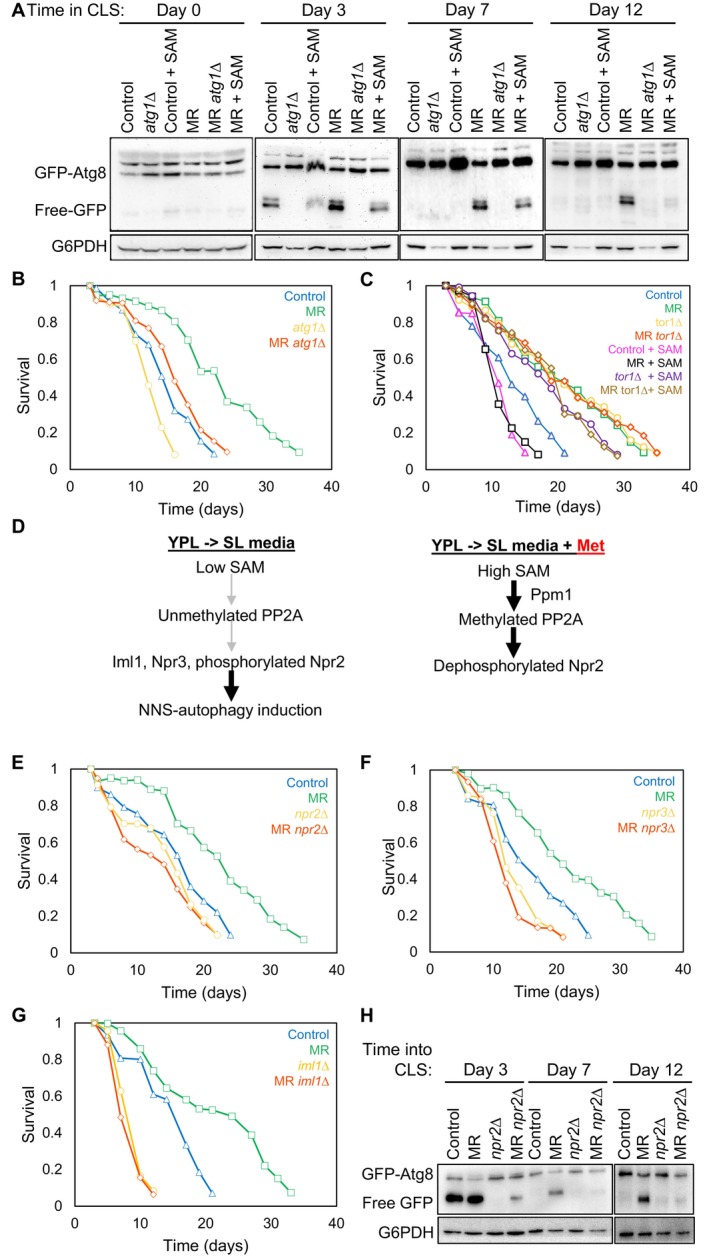
MR activates NNS‐induced autophagy to extend CLS. (A) GFP‐Atg8 western blot of control and MR samples +/− SAM (400 μM) 5 h (Day 0, still in log‐phase growth), and days (3, 7, and 12) into chronological lifespan. (B) CLS of the indicated strains. (C) CLS of the indicated strains +/− SAM (400 μM). (D) Model of NNS‐induced autophagy in yeast switched from rich, YPL media, to minimal SL media (Sutter et al. 2013). Briefly, switching from rich to minimal media with lactate as carbon source results in lowering of methionine levels (left side). This leads to less PP2A methylation, which subsequently leads to less dephosphorylation of Npr2. Phosphorylated Npr2 results in NNS‐autophagy induction. When methionine is added (right side), PP2A methylation is increased, Npr2 is dephosphorylated and activation of NNS‐induced autophagy is ceased. (E–G) CLS of the indicated strains. (H) Western blot of GFP‐Atg8 in the indicated strains at 3 days, 7 days, and 12 days into chronological lifespan.

Inspired by the fact that MR‐mediated CLS extension in yeast largely depends on autophagy (Johnson and Johnson 2014; Plummer and Johnson 2019), we directed our attention to the role of autophagy in lifespan extension. To investigate the relationship between SAM and autophagy during MR‐mediated lifespan extension, we used the GFP‐Atg8 autophagy assay to measure autophagic flux (Nair et al. 2011). Autophagy was induced, as seen by cleavage of GFP‐Atg8 to release free‐GFP, at early times (3 days) of the CLS in control strains but was inactivated by 7 days into the CLS (Figure 2A). By contrast, MR led to higher levels of autophagy compared to controls 3 days into the CLS and MR maintained autophagy flux at 7 and 12 days into the CLS (Figure 2A). Importantly, MR did not activate autophagy in cells growing in log phase (“Day 0 cells”) (Figure 2A) (Ruckenstuhl et al. 2014). These results show that MR by *MET15* deletion is not sufficient to induce autophagy (i.e., at Day 0), and that additional events occurring during chronological aging are required to induce autophagy upon MR.

Importantly, autophagy induced by MR during chronological aging was attenuated by supplementation of SAM, indicating that MR promotes autophagy induction through reducing levels of SAM (Figure 2A). Accordingly, deleting the gene encoding the key autophagy protein Atg1 blocked autophagy induction by MR during the CLS and greatly reduced CLS extension by MR (Figure 2A,B). It has previously been suggested that Tor1 inhibition—also known to extend lifespan—and MR function in the same pathway to extend chronological lifespan (Ruckenstuhl et al. 2014). We confirmed the epistatic relationship (Figure 2C) and observed that the addition of SAM to either *tor1∆* cultures or methionine restricted *tor1∆* cultures reduced, but did not prevent, CLS extension (Figure 2C).

A type of autophagy that does not require nitrogen starvation, termed non‐nitrogen‐starvation (NNS)‐induced autophagy (Sutter et al. 2013), can be achieved in non‐aging cultures following a switch from rich (YPL) media to minimal (SL) media that lacked amino acids (Figure 2D). Lack of methionine in the media leads to reduced methylation of protein phosphatase PP2A—rendering PP2A inactive against the substrate Npr2 (Sutter et al. 2013). The resulting increased phosphorylation of the Npr2 component of the SEACIT complex allows Npr2 to interact with the other components (Npr3, Iml1) of the SEACIT complex and activate NNS‐induced autophagy (Sutter et al. 2013). Induction of autophagy was halted by the addition of methionine to the SL media indicating that it is the lack of methionine that was activating NNS‐induced autophagy (Figure 2D). While the SEACIT complex is required for the inhibition of Tor1 in response to amino acid starvation (Neklesa and Davis 2009), switching from rich (YPL) media to minimal (SL) media that lacked amino acids induced NNS‐autophagy in a manner that was not accompanied by inhibition of Tor1 (apparent by no reduction in phosphorylation of the Tor1 kinase substrate Atg13) suggesting that SEACIT functions in a parallel manner or downstream of Tor1 and Atg13 to activate autophagy during methionine starvation (Sutter et al. 2013) (Figure 2D). To determine whether NNS‐induced autophagy was activated during chronological aging upon MR and whether activation of this type of autophagy is responsible for lifespan extension by MR, we deleted *NPR2* and found that this prevented CLS extension by MR (Figure 2E). Methionine‐restricted yeast without the other components of the SEACIT complex, Iml1, and Npr3, also no longer had an extended CLS (Figure 2F,G). MR was no longer capable of inducing autophagy during chronological aging in cells deleted for *NPR2* (Figure 2H). Additionally, the autophagy that is induced at Day 3 of the CLS even in control cells depended on Npr2 (Figure 2H), indicating that Npr2 is involved in activating the transient autophagy that occurs during the entry into the CLS. Note that deletion of *NPR2* does not prevent activation of nitrogen‐starvation induced autophagy (Wu and Tu 2011). Consistent with the failure to see Tor1 inhibition during NNS‐induced autophagy upon methionine starvation (Sutter et al. 2013), the level of autophagy induced by MR during the CLS is much lower than that induced by inhibition of Tor1 by 200 mM rapamycin (Figure S3A). Together, these results demonstrate that MR activates NNS‐induced autophagy, but only during the CLS, not in non‐aging cells. In agreement, MR does not inhibit Tor1 in non‐aging cells as measured by Atg13 phosphorylation levels (Figure S3B). Importantly, these studies show that MR extends the CLS by activating NNS‐induced autophagy.

### 3.4 RLS Is Extended in a Manner Dependent on Autophagy in MR Yeast

**FIGURE 3:**
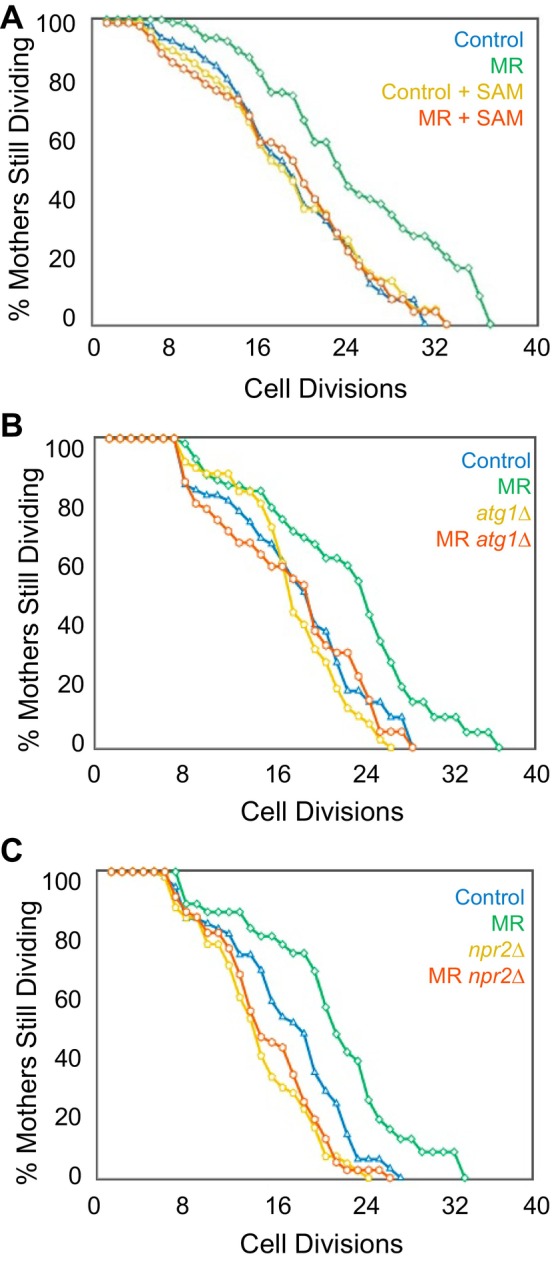
RLS is extended in a manner dependent on SAM and autophagy in MR yeast. (A) RLS of control (100 cells) and MR (*met2∆*) (100 cells) yeast strains +/− SAM (400 μM). *p* values are: Control vs. MR = 0, Control vs. Control +SAM = 0.647, Control vs. MR + SAM = 0.5555, Control +SAM vs. MR = 0, Control + SAM vs. MR+ SAM = 0.9516, MR vs. MR + SAM = 0. (B) RLS of Control (80 cells), MR (*met2∆*) (80 cells), *atg1∆* (80 cells), and MR (*met2∆*) *atg1∆* (80 cells) yeast strains; *p* values are: Control vs. MR = 0.0052, Control vs. *atg1∆* = 0.7741, Control vs. MR *atg1∆* = 1, *atg1∆* vs. MR = 0.000018, *atg1∆* vs. MR *atg1∆* = 0.1, MR vs. MR *atg1∆* = 0.0009. (C) RLS of Control (60 cells), MR (60 cells), *npr2∆* (60 cells), and MR *npr2∆* (60 cells); *p* values are: Control vs. MR = 0.0024, Control vs. *npr2∆* = 0.0078, Control vs. MR *npr2∆* = 0.0404, MR vs. *npr2∆* = 0, MR vs. MR *npr2∆* = 0, *npr2∆* vs. *npr2∆* MR = 1.

We asked whether MR can also extend the replicative lifespan (RLS) of yeast, and if so, whether it does so in a manner dependent on activation of NNS‐induced autophagy. Deletion of *MET15* did not lead to an extended RLS (Figure S4A), as it did in the CLS (Figure 1B). *met15∆* strains are only semi‐auxotrophic for methionine synthesis while deletion of *MET2*, which completely ablates methionine biosynthesis (Thomas and Surdin‐Kerjan 1997), extended the RLS (Figure 3A). However, *met2∆* cultures extended CLS the same amount as *met15∆* cultures (Figure S4B), suggesting that extension of the RLS may require stricter MR than extension of the CLS (Ruckenstuhl et al. 2014). Like the situation with extension of the CLS by MR, we found that addition of SAM fully ameliorates the RLS‐extending effect of MR (Figure 3A). Further, deletion of *ATG1* prevented MR from extending the RLS, indicating that autophagy is necessary for MR to extend the RLS (Figure 3B). Moreover, Npr2 was necessary for MR to extend the RLS (Figure 3C), suggesting that NNS‐induced autophagy also plays a role in MR's ability to extend the RLS, in addition to its requirement for CLS extension by MR (Figure 2E). These results establish that MR can extend the yeast RLS and that it does so in a manner dependent on SAM reduction, Npr2, and autophagy.

### 3.5 Methylation of PP2A Is Decreased During MR

**FIGURE 4:**
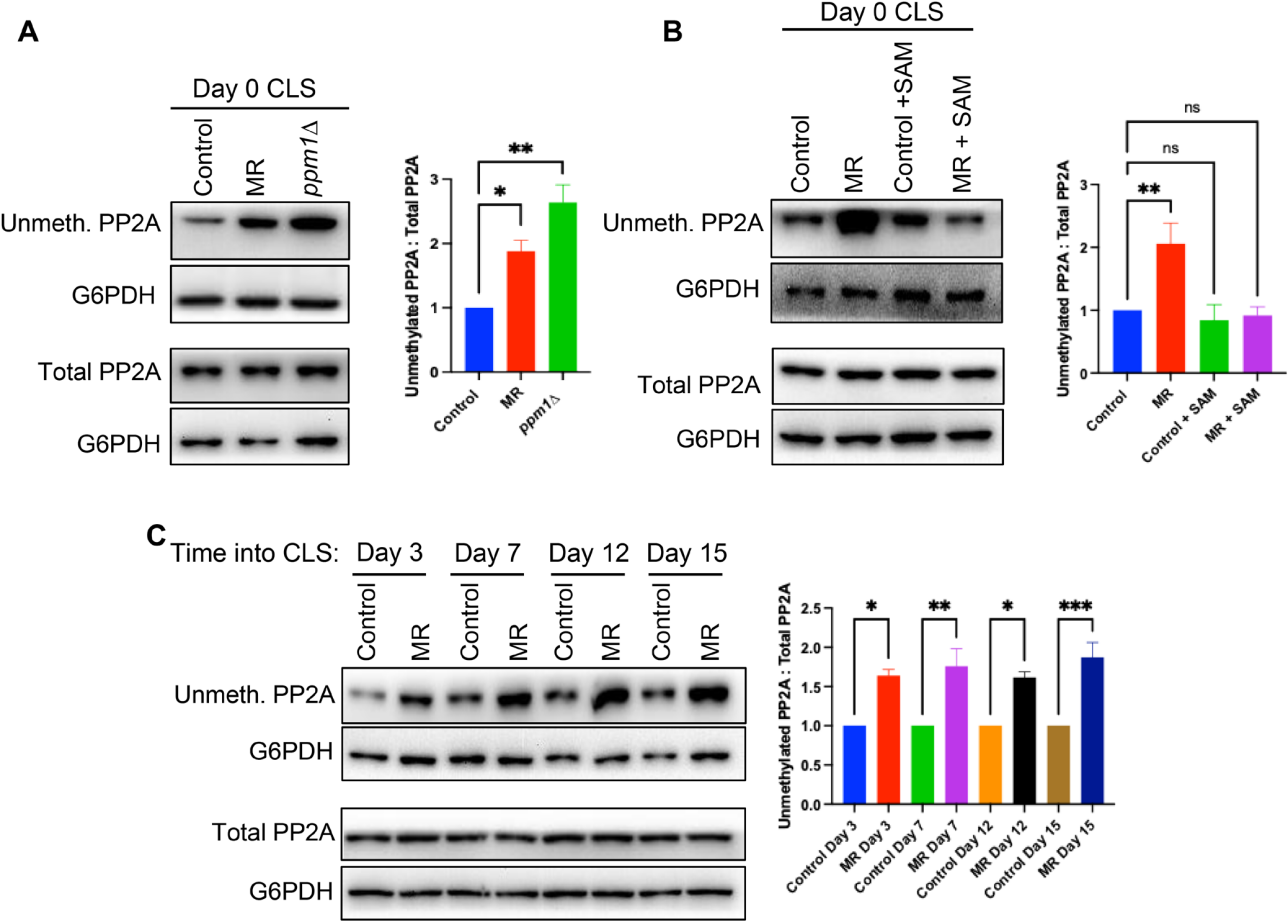
Methylation of PP2A is decreased during MR. (A) Western blot and statistical analysis of unmethylated PP2A levels 5 h into CLS in control, MR and *ppm1∆* yeast. Data are presented as the mean ± SEM (*n* = 3). Statistical significance determined by one way ANOVA comparing all experimental groups to the control; *p < 0.05, **p < 0.005. (B) Western blot and statistical analysis of unmethylated PP2A levels 5 h into CLS in control and MR yeast +/− SAM (400 μM) as in panel (A) **p < 0.005. (C) Western blot and statistical analysis of unmethylated PP2A levels on days 3, 7, 12, and 15 of CLS in control and MR yeast as in panel (A). *p < 0.05, **p < 0.005, ***p < 0.0005.

Due to SAM's role as the methyl donor for the cell, it seemed likely that MR may be increasing lifespan through reducing methylation of a methyltransferase substrate(s). Given that demethylation of PP2A is crucial for activation of NNS‐induced autophagy (Figure 2D) (Sutter et al. 2013), and knowing that components of the SEACIT complex needed for NNS‐induced autophagy were necessary for CLS extension by MR (Figure 2E–G), we directed our focus to PP2A methylation. PP2A methylation is inferred by using an antibody that recognizes only unmethylated PP2A in comparison to the total levels of PP2A (Ye et al. 2019). The same blots were stripped with NaOH to remove the methylation and reprobed with the same antibody to measure total PP2A levels. We found that MR led to less PP2A methylation compared to control yeast that had not entered the CLS (Figure 4A). As a positive control, deletion of the PP2A methyltransferase (Sutter et al. 2013), *PPM1*, led to an even greater reduction in PP2A methylation (Figure 4A). Addition of SAM back to the cultures undergoing MR blocked the reduction of PP2A methylation (Figure 4B). This reduction in PP2A methylation in cells undergoing MR was also observed on CLS days 3–15 (Figure 4C). Taken together, these results show that MR leads to reduced PP2A methylation.

Even though deletion of *PPM1* led to an even greater reduction in PP2A methylation than MR (Figure 4A), yeast lacking *PPM1* did not have an extended CLS (Figure S5A). Furthermore, deletion of *PPM1* prevented MR‐mediated lifespan extension (Figure S5A), potentially indicating that too little PP2A methylation may be detrimental to the cell due to the duplicitous roles of the phosphatase; PP2A dephosphorylates many other substrates besides Npr2. For example, methylated PP2A dephosphorylates the histone 3 lysine 36 demethylase, Rph1 and demethylation of PP2A leads to greater phosphorylation of Rph1 which results in specifically less H3K36me3 (Ye et al. 2019). Though MR leads to less PP2A methylation, we did not find Rph1 to be necessary for CLS or RLS extension by MR (Figure S5B,C). Further, since MR didn't lead to less H3K36me3, quite the opposite (Figure S2A,B), it can be inferred that MR's reduction of PP2A methylation is insufficient to impact histone methylation. These results suggest that MR establishes a beneficial ‘set‐point’ of partial PP2A demethylation that is sufficient for Npr2 phosphorylation but avoids detrimental off‐target effects that arise from the total loss of PP2A methylation upon deletion of *PPM1*.

### 3.6 Phosphorylation of Npr2, the NNS‐Induced Autophagy Regulator, Is Increased in a SAM‐Dependent Manner by MR During Chronological Aging

**FIGURE 5:**
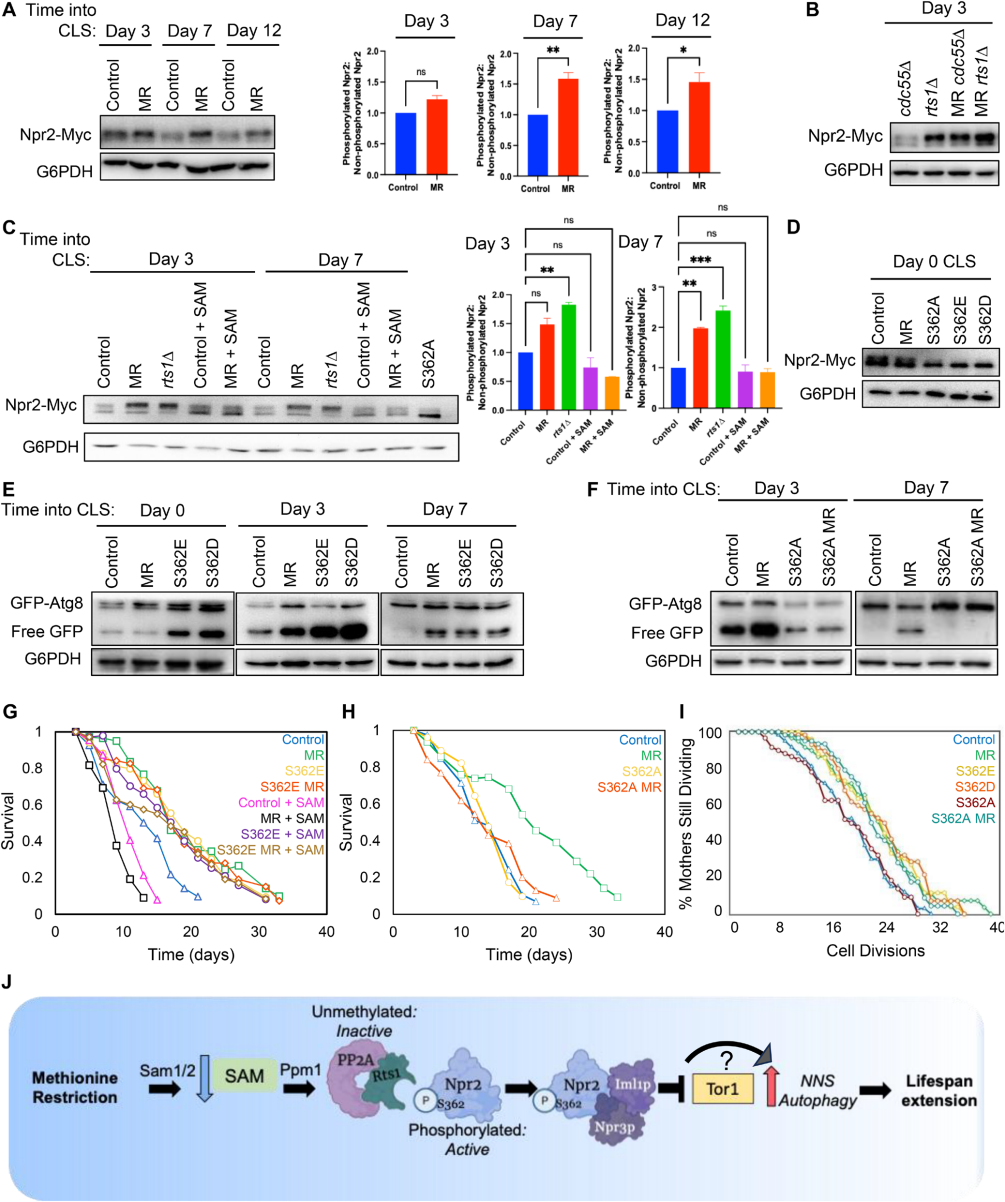
Phosphorylation of Npr2 is increased in a SAM‐dependent manner during MR and is necessary for CLS extension. (A) Western blot and statistical analysis of Npr2 phosphorylation levels on days 3, 7, and 12 of CLS in control and MR yeast. Relative Npr2 phosphorylation levels are a ratio of each sample's phosphorylated band to the unphosphorylated band. Data are presented as the mean ± SEM (*n* = 3). Statistical significance determined by one way ANOVA comparing all experimental groups to the control; *p < 0.05, **p < 0.005. (B) Western blot of Npr2 phosphorylation levels in strains lacking the two PP2A beta subunits, Cdc55 and Rts1, at day 3 of the CLS. (C) Western blot and statistical analysis of Npr2 phosphorylation levels 3 and 7 days into CLS as in panel (A). **p < 0.005, ***p < 0.0005. (D) Western blot of Npr2 phosphorylation levels during log phase growth in the Npr2 phosphomutants. (E, F) GFP‐Atg8 western blot in the indicated strains. (G) CLS of the indicated strains +/− SAM (400 μM). (H) CLS of the indicated strains. (I) RLS of Control (*n* = 60), MR (*met2∆*) (*n* = 60), Npr2 S362D (*n* = 60), Npr2 S362E (*n* = 60), Npr2 S362A (*n* = 60), and Npr2 S362A MR (*met2∆*) (*n* = 60) yeast strains. *p* values are: Control vs. MR = 0.026, Control vs. S263E = 0.0174, Control vs. S362D = 0.0079, Control vs. S362A = 1, Control vs. S362A MR = 0.023, MR vs. S362E = 1, MR vs. S362D = 1, MR vs. S362A = 0.0353, MR vs. S362A MR = 1, S362E vs. S362D = 1, S362E vs. S362A = 0.0275, S362E vs. S362A MR = 1, S362D vs. S362A = 0.0168, S362D vs. S362A MR = 1, S362A vs. S362A MR = 0.0227. (J) Schematic showing model for the mechanism of lifespan restriction by MR. Briefly, MR during CLS leads to lower levels of PP2A methylation, thus leading to PP2A being unable to, with the beta subunit Rts1, dephosphorylate Npr2 and subsequently increases Npr2 phosphorylation in non‐dividing cells. This increased phosphorylation at serine 362 leads to the activation of NNS‐induced autophagy, presumably by the Npr2‐Npr3‐Iml1 complex. We have not established whether MR‐mediated lifespan extension is via Tor1 inhibition or via a parallel, or downstream, pathway.

One of the many dephosphorylation substrates of methylated PP2A is Npr2 (Sutter et al. 2013). Due to our observation that Npr2 is necessary for CLS and RLS extension by MR (Figures 2G and 3C), and that MR decreases PP2A methylation (Figure 4), we asked whether Npr2 phosphorylation is increased during aging. Indeed, MR led to increased Npr2 phosphorylation at day 3, 7, and 12 of the CLS (Figure 5A). However, MR is not sufficient for increased Npr2 phosphorylation as the day 0 cells (non‐aging) did not show higher levels of Npr2 phosphorylation (Figure S6A). This is consistent with the fact that MR alone is not sufficient to turn on NNS‐induced autophagy (Figure 2A), indicating that the cells need to enter a chronologically aging state to observe Npr2 phosphorylation and activation of NNS‐induced autophagy.

Further, the phosphatase PP2A has two beta subunits—Cdc55 and Rts1—whose roles are to target the catalytic subunits of the phosphatase to specific substrates for dephosphorylation (Healy et al. 1991; Shu et al. 1997). Each substrate of PP2A will be targeted by one of these specific beta subunits. Deleting *RTS1*, but not *CDC55*, resulted in greater Npr2 phosphorylation (Figure 5B), indicating that Rts1 is required for dephosphorylation of Npr2. Though Rts1 increased Npr2 phosphorylation, deletion of *RTS1* did not extend the CLS (Figure S6B), perhaps because either the level of Npr2 phosphorylation is critical or increased phosphorylation of other Rts1 targeted substrates may be deleterious. There was not a combined effect of increased Npr2 phosphorylation in MR *rts1∆* cultures (Figure 5B), further indicating that Rts1 is the beta subunit of PP2A required for methionine restricted yeast to have increased Npr2 phosphorylation during chronological aging. Additionally, this increase in Npr2 phosphorylation during aging upon MR is due to decreased SAM, as addition of SAM to yeast during MR reduced Npr2 phosphorylation to a level similar to that of controls (Figure 5C).

### 3.7 MR Extends CLS, but Not RLS, Through Npr2 Phosphorylation

It was unknown which phosphorylated residue(s) on Npr2 was necessary for the formation of the SEACIT complex, and thus for activation of NNS‐induced autophagy. Mass spectrometry analysis of the entire yeast phosphoproteome identified S362 as a phosphorylated residue on Npr2 (Albuquerque et al. 2008). Thus, we made yeast strains that express phosphomimetic—S362E and S362D, or phosphodefunct‐S362A Npr2. Confirmation that this serine was indeed the phosphorylation site is provided by the fact that yeast with these three mutants only have the non‐phosphorylated Npr2 band (Figure 5D). Like MR, we found that Npr2 S362E and S362D result in prolonged autophagy flux throughout the CLS, while control cells lose autophagy after day 3 of the CLS (Figure 5E). Unlike the *met15∆* strain, mimicking Npr2 phosphorylation led to autophagic flux even in cells that have not yet entered the CLS. Importantly, the unphosphorylatable mutant, S362A, prevented MR from inducing autophagy but did not block all the autophagy that occurs at day 3 of the CLS even in control cells (Figure 5F). Moreover, mimicry of the phosphosite the S362D and S362E mutants was sufficient to extend CLS, even without MR (Figure 5G and Figure S6C). Conversely, this phosphosite is downstream of SAM because both S362E and S362D extend lifespan when supplemented with SAM (Figure 5G and Figure S6C). The unphosphorylatable mutant, S362A, prevented MR‐mediated CLS extension (Figure 5H). Like CLS, the Npr2 phosphomimetics were sufficient to extend replicative lifespan (Figure 5I). Counterintuitively, though, the S362A mutant was incapable of preventing MR's RLS extension (Figure 5I). This indicates that, while phosphorylation of Npr2 *can* induce autophagy in cells that are dividing (i.e., not in the CLS) and can extend RLS in response to MR, MR is not prolonging RLS through Npr2 phosphorylation but instead uses a different mechanism to extend the RLS dependent on Npr2 and autophagy. These results are consistent with our finding that MR is not sufficient to increase Npr2 phosphorylation in dividing cells (Figure S6A). Notably, combining MR with rapamycin treatment does not further increase the RLS extension observed with solely MR, which suggests that the two anti‐aging interventions are epistatic in their lifespan extending mechanisms (Figure S6D). Taken together, these data show that the molecular mechanism whereby MR extends lifespan in yeast entails MR‐dependent reduced PP2A methylation, which prevents the dephosphorylation of Npr2 S362 during chronological aging. This in turn allows formation of the SEACIT complex that activates NNS‐induced autophagy (Figure 5J).

### 3.8 MR at Only the Beginning of the CLS Is Sufficient to Extend Lifespan

**FIGURE 6:**
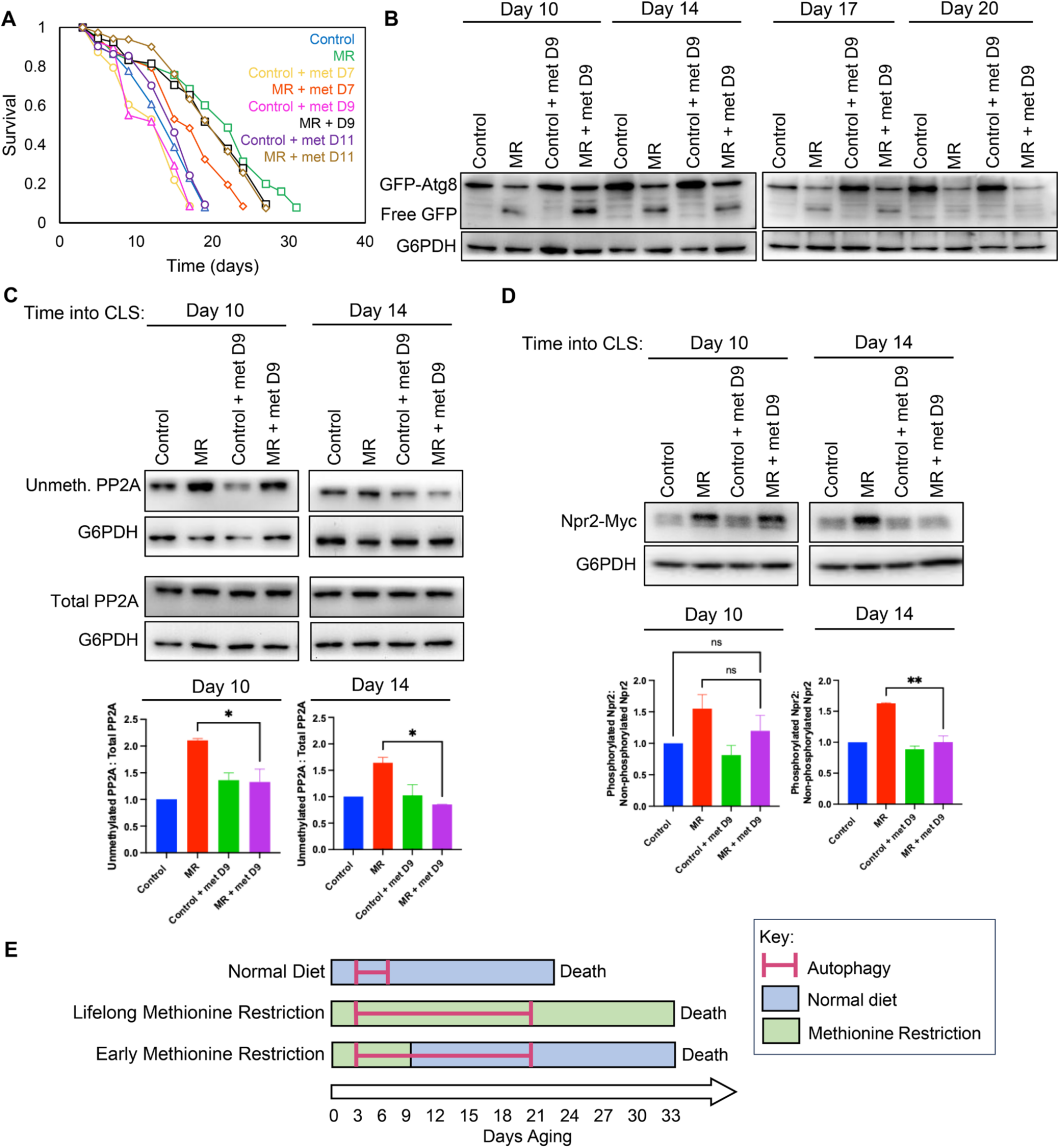
Early MR activates autophagy and extends CLS equivalently to lifelong MR. (A) CLS of Control and MR yeast +/− methionine (0.5 mM) added back to cultures on days 7, 9, and 11 of CLS. (B) GFP‐Atg8 western blot of the indicated strains +/− methionine (0.5 mM) added back to cultures on day 9. (C) Western blot and statistical analysis of unmethylated PP2A levels 10 and 14 days into CLS +/− methionine added back on day 9, as in Figure 4A. *p < 0.05. (D) Western blot and statistical analysis of Npr2 phosphorylation levels on days 10 and 14 of chronological lifespan in control and MR yeast +/− methionine added back on day 9, as in Figure 5A. **p < 0.005. (E) Schematic dictating early MR's ability to extend lifespan and promote autophagy.

Led by our observation that MR turns on autophagy beginning on day 3 of chronological lifespan, we asked whether MR only at the beginning of chronological lifespan is sufficient to extend lifespan, or whether it is continuously required. To answer this question, we added back methionine to MR cultures at days 0, 4, 7, 9, or 11 during chronological aging to end the MR. Intriguingly, though adding back methionine on days 0 and 4 prevented lifespan extension by MR (Figure S7A), cultures where methionine was added on day 7 still had a partially extended CLS, and addition of methionine on days 9 and 11 allowed for a complete extension of lifespan (Figure 6A). This suggests that restriction of methionine is only required at earlier times during chronological aging to achieve lifespan extension. In agreement, we found that autophagy persisted until day 17 of CLS regardless of whether the MR ended on day 9 or was maintained throughout the CLS (Figure 6B). Autophagy flux was largely turned off by day 20 in all cultures (Figure 6B). In agreement with 4 days of MR not being enough to extend lifespan (Figure 6A), addition of methionine on day 4 of CLS prevented the prolonged induction of autophagy seen during MR (Figure S7B). Of note, these findings are unlikely a consequence of older cells being incapable of taking up methionine due to decreased methionine transport machinery, because day 9 yeast have the same levels of Mup1—the primary methionine transporter (Isnard et al. 1996)—as day 4 yeast (Figure S7C).

Next, we examined the impact that stopping MR on day 9 had on vital components of the NNS‐induced autophagy pathway. Adding back methionine on day 9 increased PP2A methylation in MR yeast by day 10, and it reduced Npr2 phosphorylation by day 14 (Figure 6C,D), providing evidence for methionine uptake not being defective in the older cells. In all, these data collectively indicate that, while stopping MR after 9 days of chronological aging results in the subsequent reversal of the MR‐induced post‐translational modification of components of the NNS‐induced autophagy pathway, the autophagy and lifespan extension persist to the level seen with constant MR. From this, we have crafted a new schema for early MR, whereby yeast cells harness a memory for autophagy induction long after methionine is added back to the cells and the signaling pathway that activated the NNS‐induced autophagy pathway is turned off (Figure 6E). Overall, this demonstrates that transient MR treatment in early life is a novel longevity intervention, suggesting that aging may be targeted already in younger cells.

## 4 Discussion

Here, we have uncovered the molecular mechanism whereby MR extends the lifespan of budding yeast. This mechanism entails lowered levels of the methyl‐donor SAM, as a consequence of methionine restriction, resulting in reduced levels of methylated protein phosphatase PP2A. Coupled with the induction of Npr2 phosphorylation that occurs during chronological aging, the inability of the unmethylated PP2A to dephosphorylate Npr2 during MR leads to the activation of NNS‐induced autophagy during chronological aging, resulting in lifespan extension. Moreover, MR only early in lifespan is sufficient to turn on autophagy throughout the lifespan, extending lifespan to the same degree as persistent MR.

### 4.1 MR Activates NNS‐Induced Autophagy During Chronological Aging but Not During Normal Growth

From our collective data, we propose a model for the fundamental molecular mechanism by which methionine restriction extends lifespan in yeast (Figure 5J). Methionine is processed into the universal methyl donor SAM, and we found that the mechanism whereby MR extends lifespan is solely due to the reduced levels of SAM during MR, because supplementation of SAM, but not its downstream product SAH, blocked extension of both CLS and RLS by MR (Figures 1 and 3). Other methyl acceptors that have been previously implicated in aging do not appear to be influenced by MR during chronological aging in yeast; histones did not have strongly reduced methylation during MR (Table S1), and yeast do not have DNA methylation. Rather, our studies reveal that the critical factor with reduced methylation during MR that influences lifespan is likely protein phosphatase 2A (PP2A). Whether the methyltransferase Ppm1 that methylates PP2A has a lower affinity for SAM than the histone methyltransferases is unknown.

The degree of methionine restriction that extends the chronological lifespan lowered PP2A methylation levels—regardless of whether cells are in a non‐dividing chronologically aging state or not (Figure 4) only triggered Npr2 phosphorylation and NNS‐induced autophagy during chronological aging (Figures 2 and 5). Methylated PP2A is known to dephosphorylate Npr2, preventing it from inducing NNS‐induced autophagy (Sutter et al. 2013). However, the decrease in PP2A methylation during MR is not accompanied by increased levels of Npr2 phosphorylation or NNS‐induced autophagy in non‐aging cultures (Figure S6A and Figure 5D). This is presumably because Npr2 is not significantly phosphorylated during MR in non‐aging cultures rendering it irrelevant whether the Npr2 phosphatase is active or not. As such, the events induced by the degree of MR that extends lifespan are quite distinct from the original description of NNS‐induced autophagy, where a switch from rich to minimal nutrient medium was sufficient for loss of methylation of PP2A, phosphorylation of Npr2, and induction of NNS‐induced autophagy (Sutter et al. 2013). By contrast, our data indicate additional changes to the metabolic environment that occur during chronological aging are required in addition to MR to activate NNS‐induced autophagy to extend lifespan.

What is the event that synergizes with MR during chronological aging to activate NNS‐induced autophagy? We propose that as cells enter the CLS (day 3) a kinase, likely Casein Kinase 1 or 2 (Yck1 or Yck2) (Spielewoy et al. 2010), is induced to phosphorylate Npr2. While the Npr2 is efficiently dephosphorylated by methylated PP2A in cells that are methionine replete, in cells undergoing MR the levels of Npr2 phosphorylation increased early in the CLS (Figure 5A) presumably due to the unmethylated PP2A—and its beta subunit Rts1—not being able to target Npr2 for dephosphorylation. Once phosphorylated, Npr2 associates with Npr3 and Iml1 in the SEACIT complex (Ye et al. 2019) and induces NNS‐induced autophagy (Sutter et al. 2013). We show that the vital phosphorylated residue on Npr2 is S362, and that without this phosphorylation, MR is no longer able to activate NNS‐induced autophagy and extend chronological lifespan.

We further show that MR leads to persistent autophagy flux during CLS. Autophagy is known to play an integral role in antiaging by other interventions; extension of the yeast CLS by treatment with the polyamine spermidine and rapamycin require induction of autophagy (Alvers et al. 2009; Eisenberg et al. 2009). While autophagy has been shown to be important for chronological lifespan extension by MR before (Ruckenstuhl et al. 2014), we show that MR specifically induces NNS‐induced autophagy, and that this autophagy is prolonged until around day 20 of lifespan—about 17 days longer than methionine replete cells. On day 3, which is post‐diauxic shift, both methionine replete and MR yeast experience an increase in autophagic flux (Figure 2A). Stationary phase is a point of nutrient stress for the cell, and it triggers a stress response that results in the activation of many survival pathways—including autophagy (Herman 2002). So, while the increase in autophagy is not unique to the MR yeast during early times during chronological aging, it is only maintained in MR yeast at later times in the CLS.

### 4.2 Molecular Mechanism of the Extension of the Replicative Lifespan by MR

We show here that MR can extend the RLS. Moreover, MR utilizes a similar but non‐identical molecular mechanism to extend RLS and CLS. Notably, more profound MR appears to be required for extension of the lifespan by MR compared to that required for extending chronological lifespan. The reason for this is not clear but may be related to the fact that the additional hormetic stresses, including autophagy, that are induced as cells enter chronological aging are absent from replicative aging. We found that MR extends the RLS through decreasing levels of SAM and inducing autophagy in a manner dependent on Npr2. However, the complete molecular mechanism of autophagy induction and RLS extension by MR may not be identical to that occurring during CLS, because even though the RLS extension can be mimicked by phosphorylated Npr2 (Figure 5I), RLS extension via MR was not blocked by a mutation that prevents Npr2 phosphorylation (Figure 5I). It will be interesting to define the basis for this difference between MR‐induced CLS and RLS extension in the future.

### 4.3 MR in Early Lifespan Is Sufficient to Extend Chronological Lifespan

We have discovered that MR only in the beginning of chronological lifespan (day 0–9) is sufficient to prolong autophagy and extend lifespan. Intriguingly, although autophagy flux with only early MR exposure was just as persistent through the lifespan as with continuous MR, we find that cells undergoing only early MR show increased methylation of PP2A and subsequent loss of phosphorylation of Npr2 after the MR phase was ended. This raises the question of how autophagy is maintained late in the CLS following early MR in the absence of phosphorylated Npr2? One proposed model could be related to the fact that Tor1 inhibition upon nutrient stress or nitrogen starvation leads to the dephosphorylation and subsequent translocation of the transcriptional regulator Rim15 to the nucleus (Devenish and Prescott 2015). Rim15 is responsible for phosphorylating the histone demethylase Rph1 which is a transcriptional repressor of ATG genes (Bernard et al. 2015). When phosphorylated, Rph1 is no longer able to bind to DNA and cannot repress ATG genes, thus leading to induction of autophagy (Bernard et al. 2015). Indeed, Rph1 is fully phosphorylated at day 3 of the CLS and becomes dephosphorylated later in the CLS in methionine replete media accompanied by Rph1 binding to DNA and demethylation of H3K36me3 (Li et al. 2023). Given the consistently high levels of methylated H3K36me3 throughout the CLS during persistent MR and early MR (Figure S8), future studies should determine whether Rph1 is either not sufficiently dephosphorylated, not active enough, or not abundant enough to demethylate H3K36me3 and repress transcription of ATG genes at late times in the CLS of cells undergoing exposed to early MR to test this proposed model.

The finding that methionine restriction during the initial phase of yeast lifespan is sufficient to induce persistent autophagy and extended chronological lifespan highlights a critical time sensitive window for longevity interventions. Autophagy induction during this early period primes cells for sustained stress resistance even after methionine levels are restored. This suggests that early life nutrient signaling can establish a “longevity memory” triggering pathways like vacuolar acidification and mitochondrial maintenance that persist throughout the organism's lifespan (Ruckenstuhl et al. 2014; Tyler and Johnson 2018). Extending our finding that MR in early life is sufficient to extend yeast lifespan implies that nutrient timing—not just composition—may optimize human healthspan. Short term dietary adjustments during specific life phases might activate autophagy and stress‐response pathways to a similar extent as lifelong regimens, reducing practical barriers to compliance. With the knowledge of how MR extends lifespan at the molecular level in yeast, it will be interesting to identify any possible novel interventions for humans that promote healthspan and to determine the time‐sensitive window to optimize the beneficial effect.

## Author Contributions

K.B. performed all the experiments, with some assistance from N.A., T.G., and I.G., analyzed the data, and wrote the first draft of the manuscript. U.C. guided experiments by T.G., and J.T. oversaw the project and edited the manuscript. S.S. performed the mass spectrometry analyses.

## Funding

This work was supported by the National Institutes of Health (GM139816, AG079883, S10OD030286) and Hevolution Foundation.

## Conflicts of Interest

The authors declare no conflicts of interest.

## Supporting information


**Table S1:** Exhaustive, unfiltered quantitative data for all histone PTMs detected in mass spectrometry analysis in the following conditions and timepoints during yeast chronological lifespan: 5 h Control (*n* = 3), 5 h MR (*met15∆*) (*n* = 3), Day 3 Control (*n* = 3), and Day 3 MR (*n* = 3). Data include: area under the curve, ratio of relative modification occupancy of the PTM within its peptide group, and retention time (RT min) for each peptide.

**Figure S1:** (a) Chronological lifespan of BY4742, BY4741, and BY4742 *met15∆* strains. (b) Chronological lifespan of Control (BY4742) and CR (0.05% glucose) +/− SAM (400 μM).
**Figure S2:** (a) Histone modification LC–MS/MS in log phase cultures and yeast aged to day 3 of CLS in Control (*n* = 3) and MR (*met15∆*) (*n* = 3) yeast. Error bars represent SD. (b) Western blot of H3K36me3 levels in Control, MR (*met15∆*), and *rph1∆* in dividing yeast, and yeast aged to day 3 and day 6 of CLS.
**Figure S3:** (a) Atg8‐GFP cleavage assay of autophagy showing that the level of autophagy induced by MR late in the CLS is on par with that induced by 1 nM rapamycin, an amount that can extend the CLS, and is much less than that induced by 200 nM rapamycin, an amount that fully inhibits Tor1. (b) Phosphorylation analysis of Tor1 substrate Atg13 in log phase, dividing cells, showing that MR and 1 nM rapamycin do not noticeably reduce Atg13 phosphorylation, compared to the almost complete lack of Atg13 phosphorylation achieved by 200 nM rapamycin.
**Figure S4:** (a) RLS of Control (60 cells) and *met15∆* (60 cells); *p* value Control vs. *met15∆* = 0.183. (b) CLS of control (BY4742), *met15∆* (BY4741), and *met2∆* (BY4742) strains.
**Figure S5:** (a) CLS of Control, MR (*met15∆*), *ppm1∆*, and *ppm1∆* MR yeast. (b) CLS of Control and MR (*met15∆*) yeast in rph*1∆* strains. (c) RLS of Control, MR (*met2∆*), *rph1∆*, and MR (*met2∆*) *rph1∆* yeast strains. *p* values are: Control vs. MR = 0.0001, Control vs. *rph1∆* = 0.9516, Control vs. MR *rph1∆* = 0.0004, MR vs. *rph1∆* = 0.0002, MR vs. MR *rph1∆* = 0.9669, *rph1∆* vs. MR *rph1∆* = 0.0007.
**Figure S6:** (a) Western blot of Npr2 phosphorylation levels of log phase (5 h into CLS) yeast. (b) CLS of Control, MR (*met15∆*), *cdc55∆*, and *rts1∆* yeast strains. (c) CLS of Control, MR (*met15∆*), Npr2 S362D, and Npr2 S362D MR (*met15∆*) yeast strains +/− SAM (400 μM). (d) RLS of Control (93 cells), MR (115 cells), Rapamycin‐treated control (1 nM) (76 cells), and Rapamycin‐treated MR yeast strains (1 nM) (104 cells); *p* values are: Control vs. MR = 0, Control vs. Rapamycin = 0, Control vs. MR Rapamycin = 0, MR vs. Rapamycin = 0.2552, MR Rapamycin vs. Rapamycin = 0.1859.
**Figure S7:** (a) CLS of Control and MR (*met15∆*) yeast with methionine (0.5 mM) added back on day 0 and day 4 of CLS. (b) GFP‐Atg8 western blot to assess autophagy induction in Control and MR (*met15∆*) yeast with or without methionine (0.5 mM) added back to cultures on day 4. (c) Western blot of C terminal Myc tagged Mup1 in Control and MR (*met15∆*) yeast aged to days 4 and 9 of CLS.
**Figure S8:** Western blot of H3K36me3 levels in Control and MR (*met15∆*) yeast with and without methionine supplementation (0.5 mM) on day 9 aged to days 6, 10, and 14 of CLS.

## Data Availability

The mass spectrometry raw files are available on the online repository ProteomeXchange (PRIDE) under the project number PXD069602. Reviewers can access the dataset using the token “12Jfoc9fbDJx.” Alternatively, reviewers can access the dataset by logging in to the PRIDE website using the following account details: Username: reviewer_pxd069602@ebi.ac.uk; Password: bvnxunNJUxFH.
