# Supplementary material for: Methionine Restriction Extends Yeast Lifespan by Activating Non‐Nitrogen‐Starvation‐Induced Autophagy Through Limiting Methylation of Protein Phosphatase 2A
Source: Aging Cell. 2026 May 20;25(6):e70550. doi: 10.1111/acel.70550 (PMC13240291; doi:10.1111/acel.70550)
Supplement: Supplementary file 2 — Figure S1: (a) Chronological lifespan of BY4742, BY4741, and BY4742 met15∆ strains. (b) Chronological lifespan of Control (BY4742) and CR (0.05% glucose) +/− SAM (400 μM). Figure S2: (a) Histone modification LC–MS/MS in log phase cultures and yeast aged to day 3 of CLS in Control (n = 3) and MR (met15∆) (n = 3) yeast. Error bars represent SD. (b) Western blot of H3K36me3 levels in Control, MR (met15∆), and rph1∆ in dividing yeast, and yeast aged to day 3 and day 6 of CLS. Figure S3: (a) Atg8‐GFP cleavage assay of autophagy showing that the level of autophagy induced by MR late in the CLS is on par with that induced by 1 nM rapamycin, an amount that can extend the CLS, and is much less than that induced by 200 nM rapamycin, an amount that fully inhibits Tor1. (b) Phosphorylation analysis of Tor1 substrate Atg13 in log phase, dividing cells, showing that MR and 1 nM rapamycin do not noticeably reduce Atg13 phosphorylation, compared to the almost complete lack of Atg13 phosphorylation achieved by 200 nM rapamycin. Figure S4: (a) RLS of Control (60 cells) and met15∆ (60 cells); p value Control vs. met15∆ = 0.183. (b) CLS of control (BY4742), met15∆ (BY4741), and met2∆ (BY4742) strains. Figure S5: (a) CLS of Control, MR (met15∆), ppm1∆, and ppm1∆ MR yeast. (b) CLS of Control and MR (met15∆) yeast in rph1∆ strains. (c) RLS of Control, MR (met2∆), rph1∆, and MR (met2∆) rph1∆ yeast strains. p values are: Control vs. MR = 0.0001, Control vs. rph1∆ = 0.9516, Control vs. MR rph1∆ = 0.0004, MR vs. rph1∆ = 0.0002, MR vs. MR rph1∆ = 0.9669, rph1∆ vs. MR rph1∆ = 0.0007. Figure S6: (a) Western blot of Npr2 phosphorylation levels of log phase (5 h into CLS) yeast. (b) CLS of Control, MR (met15∆), cdc55∆, and rts1∆ yeast strains. (c) CLS of Control, MR (met15∆), Npr2 S362D, and Npr2 S362D MR (met15∆) yeast strains +/− SAM (400 μM). (d) RLS of Control (93 cells), MR (115 cells), Rapamycin‐treated control (1 nM) (76 cells), and Rapamycin‐treated MR yeast strains (1 nM) (104 cells); p v [file ACEL-25-e70550-s002.pptx]

## Slide 1
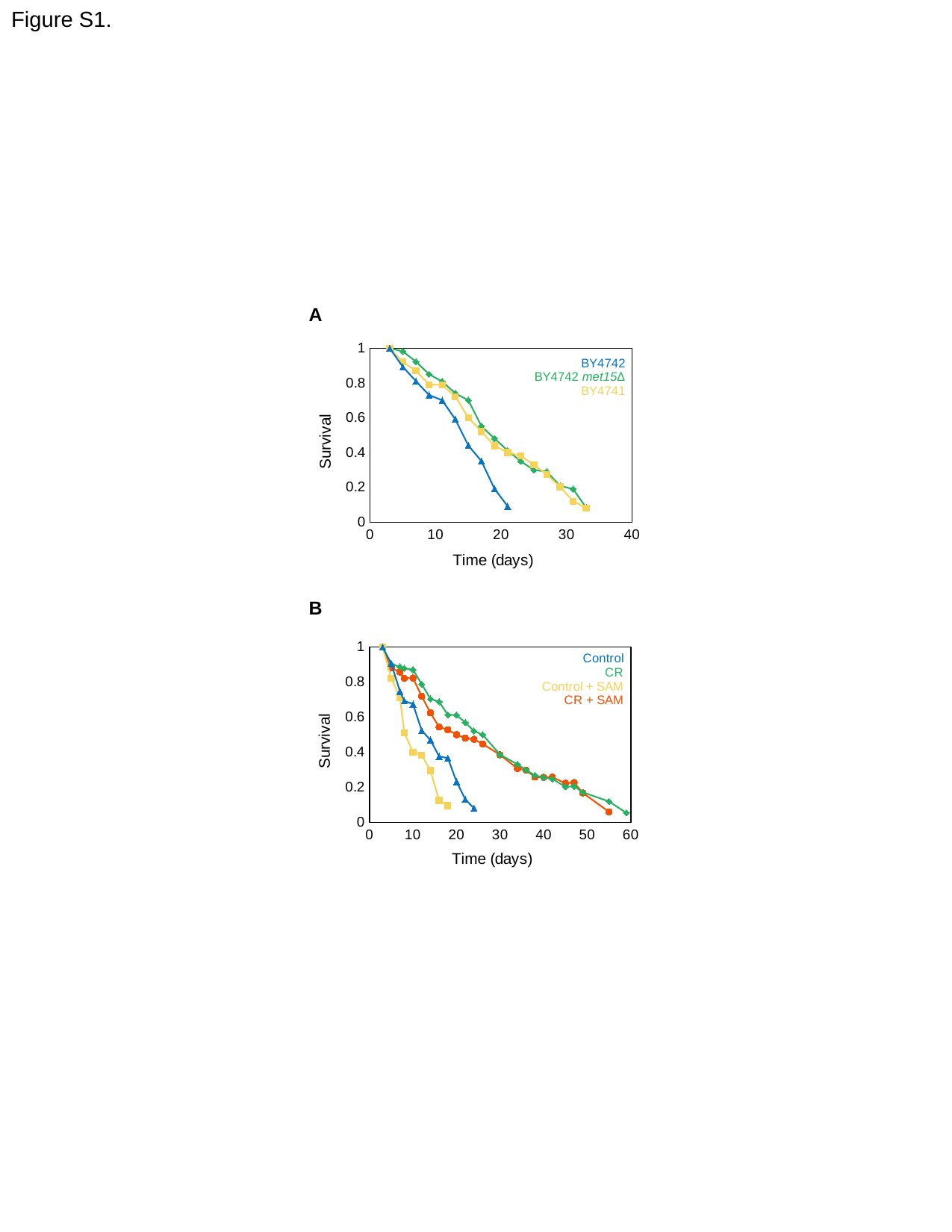

Figure S1.
A
### Chart
| Category | BY4742 | BY4742 met15∆ | BY4741 |
|---|---|---|---|B
### Chart
| Category | Control | Control+SAM | 0.05% glucose | 0.05% glucose+SAM |
|---|---|---|---|---|

## Slide 2
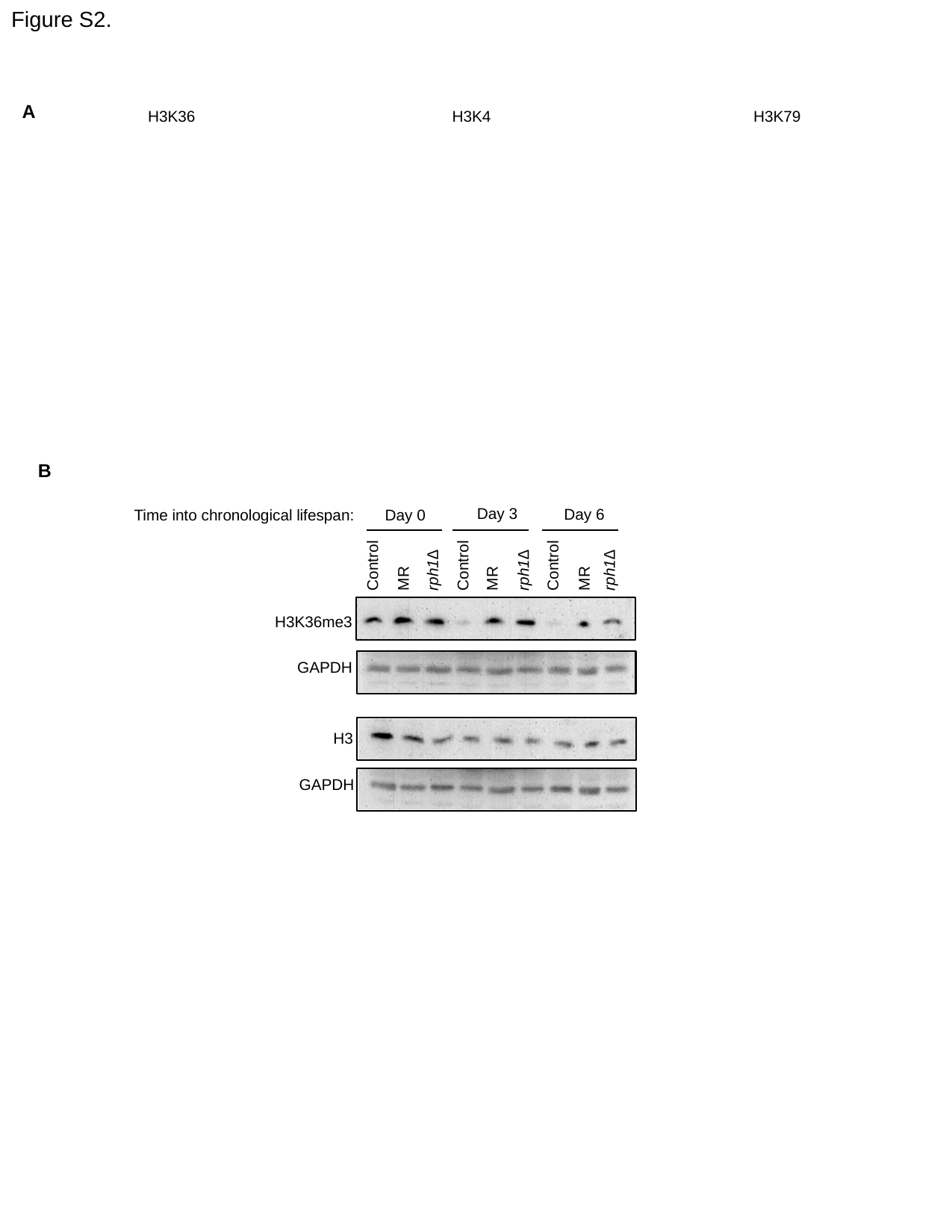

Figure S2.
A
H3K79
H3K36
H3K4
B
Day 3
Day 6
Time into chronological lifespan:
Day 0
Control
Control
Control
rph1∆
rph1∆
rph1∆
MR
MR
MR
H3K36me3
GAPDH
H3
GAPDH

## Slide 3
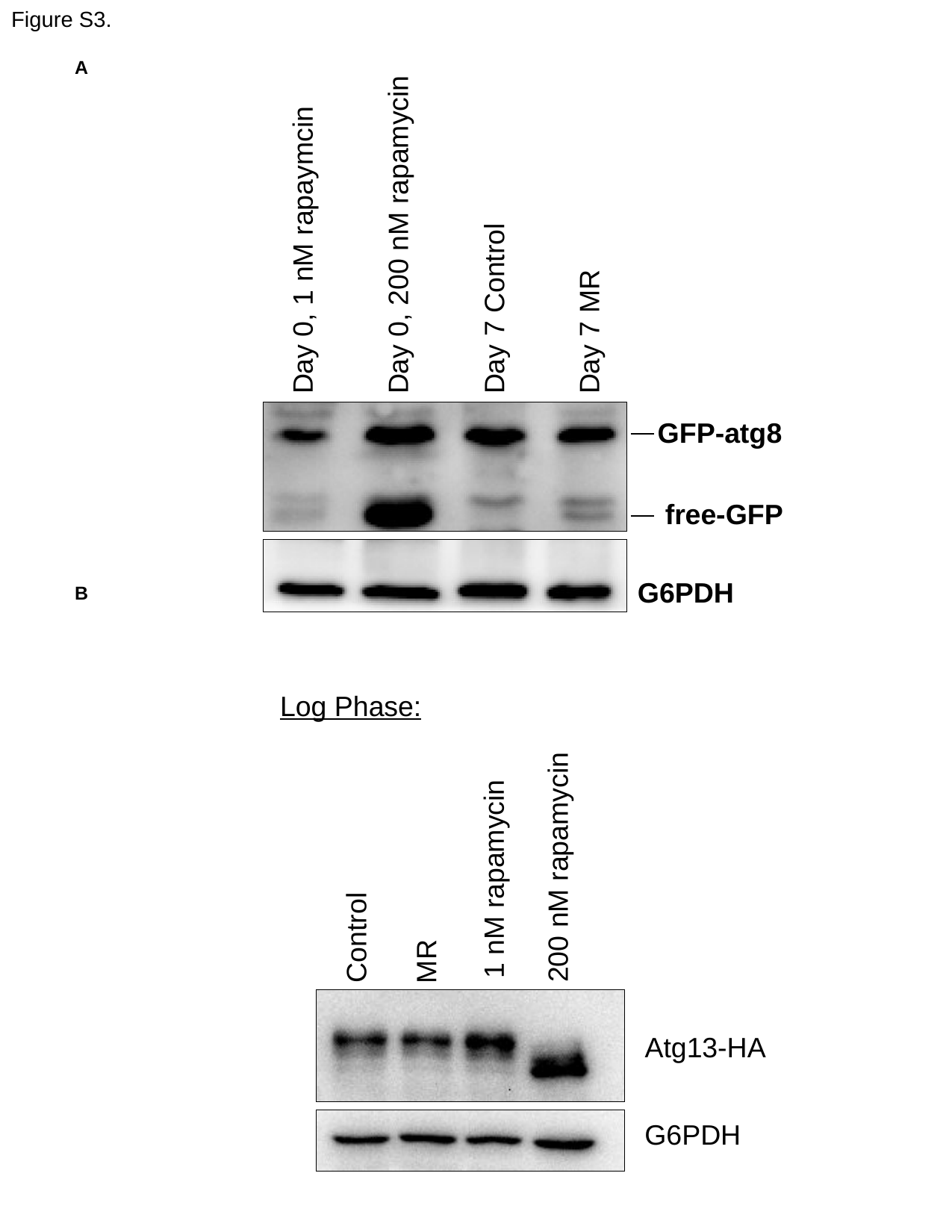

Figure S3.
A
Day 0, 200 nM rapamycin
Day 0, 1 nM rapaymcin
Day 7 Control
Day 7 MR
GFP-atg8
free-GFP
G6PDH
B
Log Phase:
200 nM rapamycin
1 nM rapamycin
Control
MR
Atg13-HA
G6PDH

## Slide 4
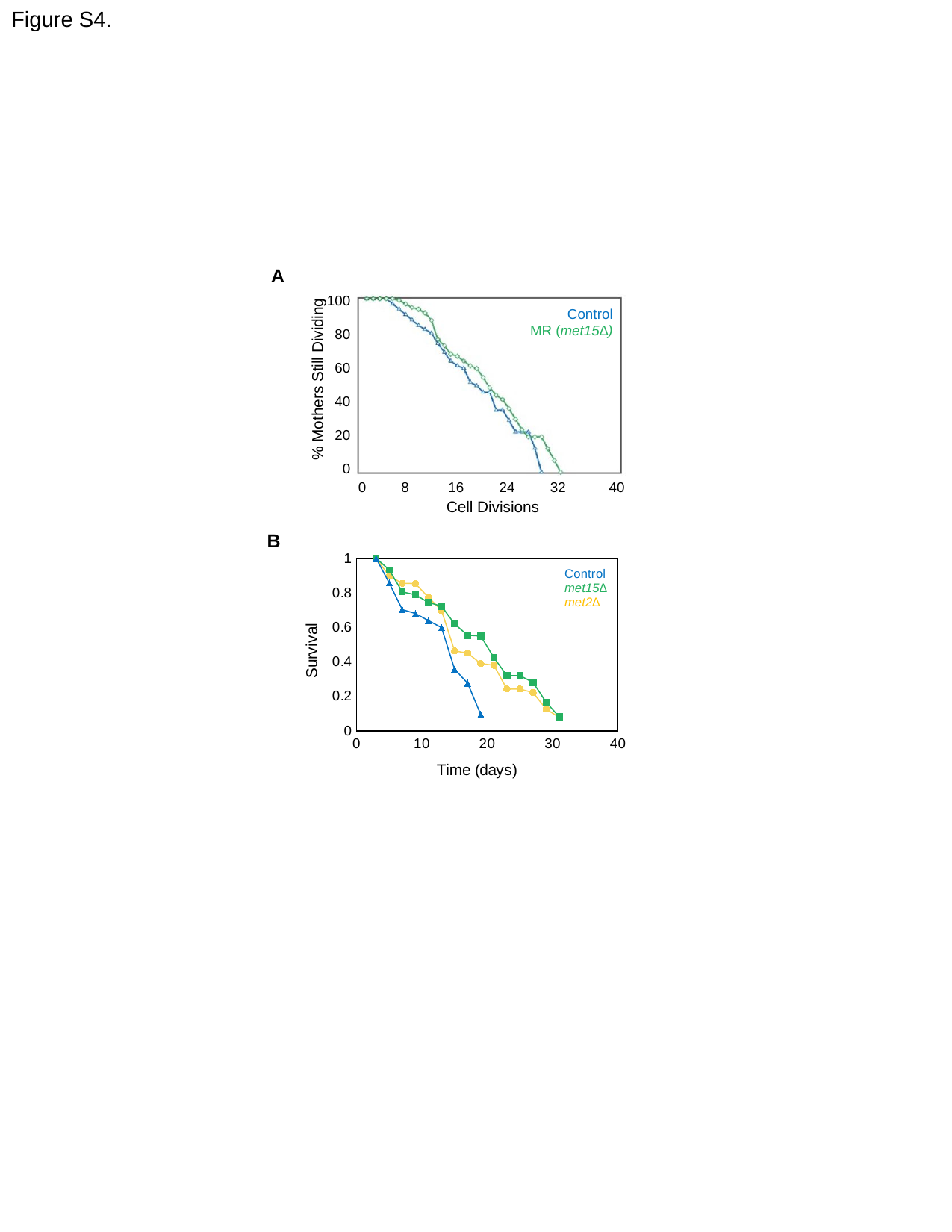

Figure S4.
A
Control
MR (met15∆)
% Mothers Still Dividing
Cell Divisions
100
 80
 60
 40
 20
 0
0 8 16 24 32 40
B
### Chart
| Category | Control | met15∆ | met2∆ |
|---|---|---|---|

## Slide 5
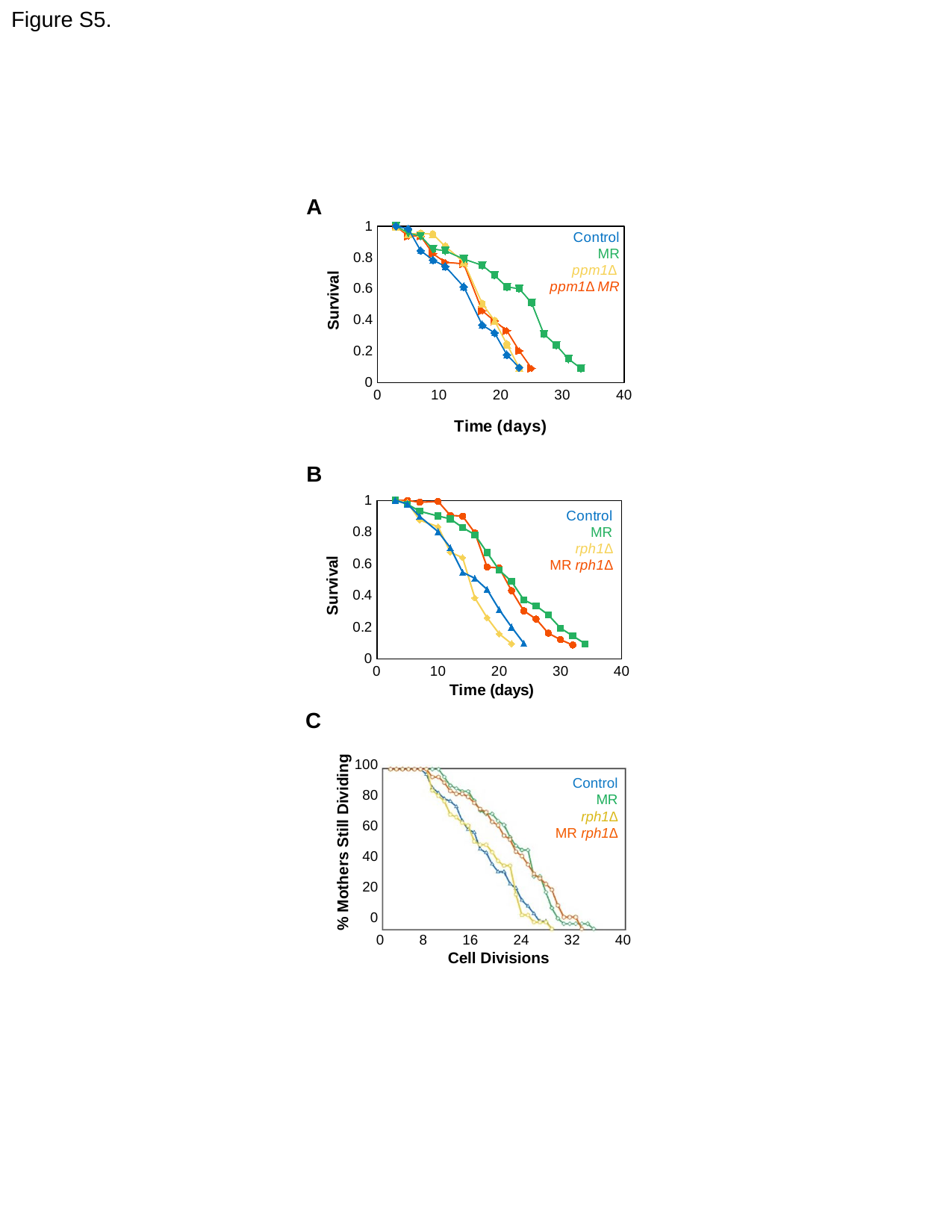

Figure S5.
A
### Chart
| Category | Control | MR | ppm1∆ | ppm1∆ MR | Control | MR | ppm1∆ | ppm1∆ MR |
|---|---|---|---|---|---|---|---|---|B
### Chart
| Category | Control | MR | rph1∆ | rph1∆ MR |
|---|---|---|---|---|C
100
 80
 60
 40
 20
 0
% Mothers Still Dividing
0 8 16 24 32 40
Cell Divisions
Control
MR
rph1∆
MR rph1∆

## Slide 6
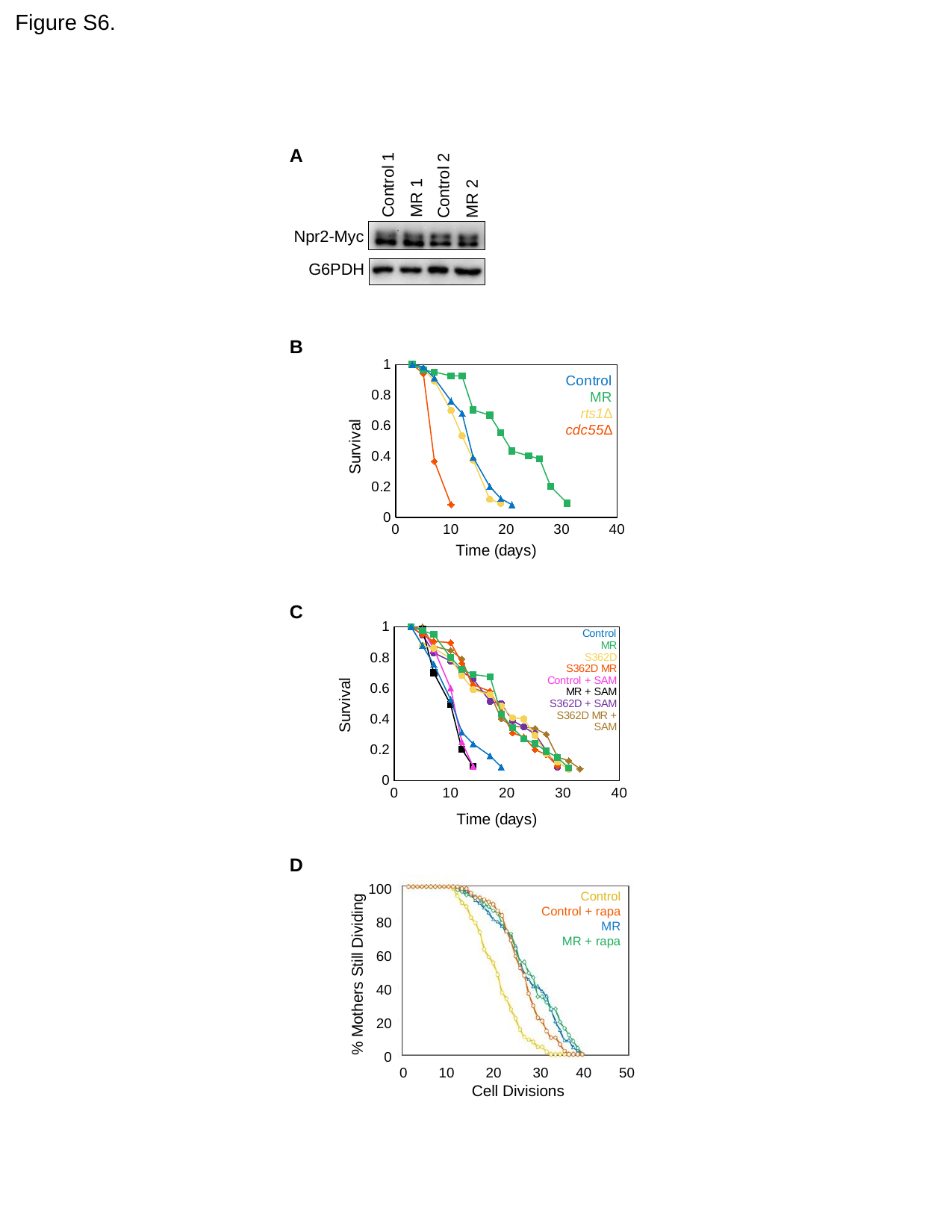

Figure S6.
A
Control 1
MR 1
Npr2-Myc
G6PDH
Control 2
MR 2
B
### Chart
| Category | Control | MR | rts1 | cdc55 |
|---|---|---|---|---|C
### Chart
| Category | Control | MR | S362D | S362D MR | Control + SAM | MR + SAM | S362D + SAM | S362D MR + SAM |
|---|---|---|---|---|---|---|---|---|D
100
 80
 60
 40
 20
 0
Control
Control + rapa
MR
MR + rapa
% Mothers Still Dividing
Cell Divisions
0 10 20 30 40 50

## Slide 7
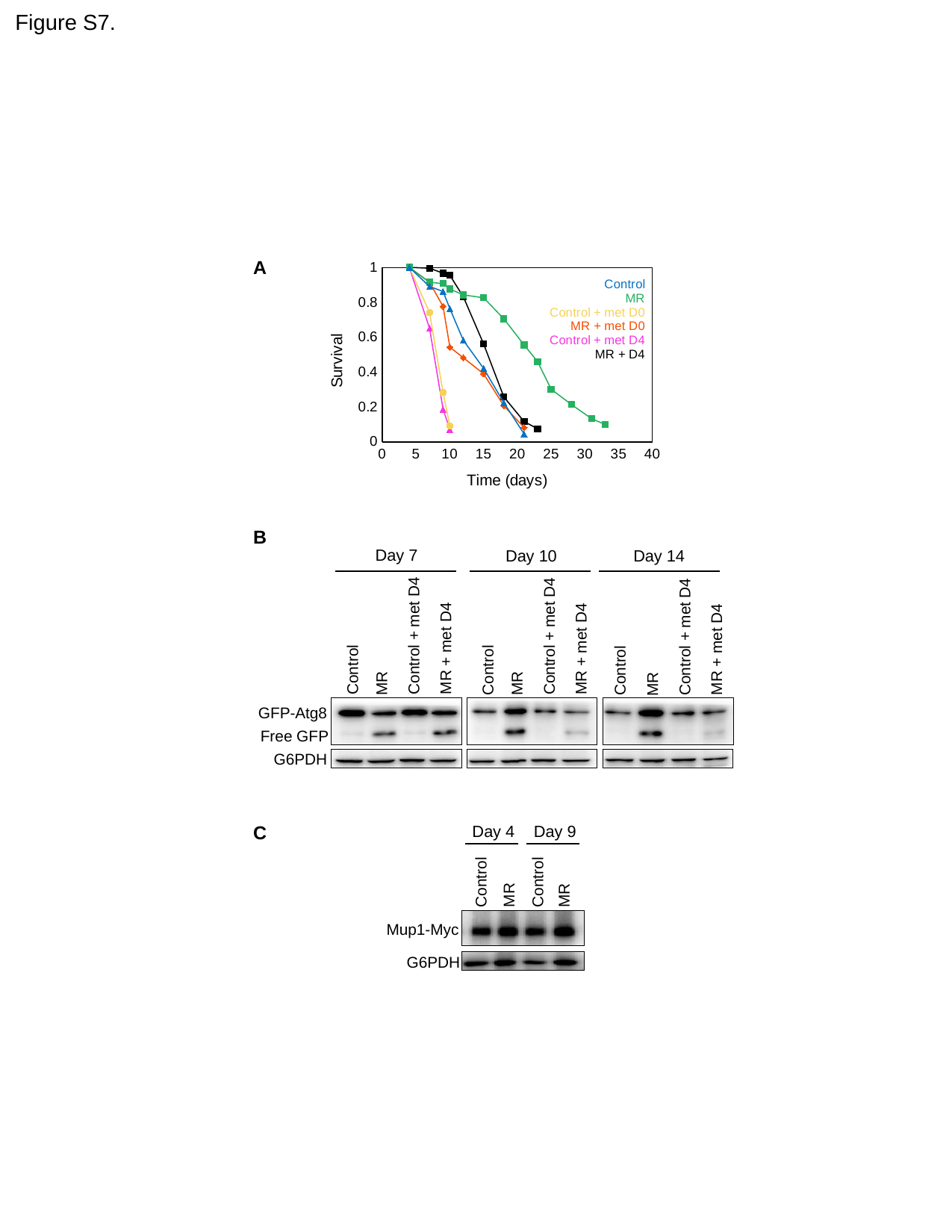

Figure S7.
A
### Chart
| Category | Control | MR | Control + met D0 | MR + met D0 | Control + met D4 | MR + met D4 |
|---|---|---|---|---|---|---|B
Day 7
Day 10
Day 14
Control + met D4
Control + met D4
Control + met D4
MR + met D4
MR + met D4
MR + met D4
Control
Control
Control
MR
MR
MR
GFP-Atg8
Free GFP
G6PDH
C
Day 4
Day 9
Control
Control
MR
MR
G6PDH
Mup1-Myc

## Slide 8
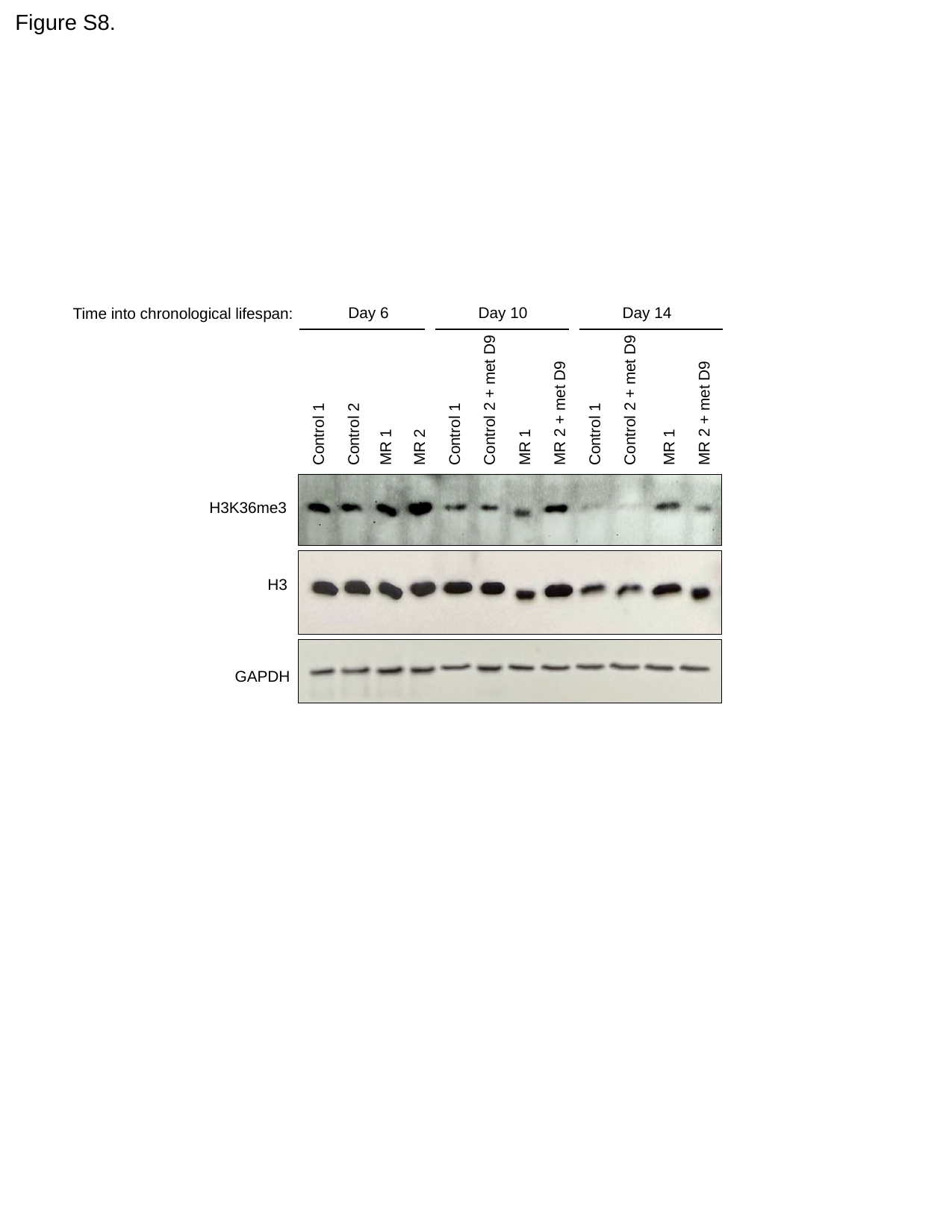

Figure S8.
Day 6
Day 10
Day 14
Time into chronological lifespan:
Control 2 + met D9
Control 2 + met D9
MR 2 + met D9
MR 2 + met D9
Control 1
Control 2
Control 1
Control 1
MR 1
MR 2
MR 1
MR 1
H3K36me3
H3
GAPDH
